# Supplementary material for: Brain morphology in Anorexia Nervosa and its subtypes: A multi-cohort study of individual participant data
Source: PLoS Med. 2026 May 20;23(5):e1004809. doi: 10.1371/journal.pmed.1004809 (PMC13215615; doi:10.1371/journal.pmed.1004809)
Supplement: S1 Appendix — (DOCX) [file pmed.1004809.s001.docx]

**Brain morphology in Anorexia Nervosa and its subtypes: A multi-cohort study of individual participant data**

**Supplementary Material**

Fabio Bernardoni¹, Dominic Arold¹^,2^, Luis Schoppik¹, Klaas Bahnsen¹^,3^, Ruiyang Ge^4^, Clara Moreau^5^, Lasse Bang^6^, Federico D’Agata^7^, Giovanni Abbate-Daga^7,8^, Christian K. Tamnes^9,10^, Iain Campbell^11,12^, Owen O’Daly^11,12^, Ulrike Schmidt^11,12^, Ph.D., Guido Frank^13,14^ , Stefanie Horndasch^15,16^, Andreas Hess^17,18,19^ Arnd Dörfler^20^ , Hans-Christoph Friederich^21^ , Joe Simon^21^, Angela Favaro^22^, Luca Lavagnino^23^ , Christina Wierenga^13,14^  , Amanda Bischoff-Grethe^13,14^, Amy E. Miles^24^ , Allan Kaplan^24^ , Aristotle Voineskos^24^, Paul A.M. Smeets²^5,^²6, Annemarie A. van Elburg^27^, Unna Danner^27^, Sophia I. Thomopoulos²^8^ , Laura Berner²^9^ , Neda Jahanshad²^8^, Sophia Frangou^29^, Joseph A. King¹, Paul Thompson²^8^, Stefan Ehrlich¹^,2^

^1^Translational Developmental Neuroscience Section, Division of Psychological and Social Medicine and Developmental Neurosciences, Faculty of Medicine, Technische Universität Dresden, Dresden, Germany

^2^Eating Disorders Research and Treatment Center, Department of Child and Adolescent Psychiatry, Faculty of Medicine, Dresden University of Technology, Dresden, Germany

^3^Maurice Wohl Clinical Neuroscience Institute, Department of Psychological Medicine, Institute of Psychiatry, Psychology and Neuroscience, King’s College London, London, United Kingdom

^4^Djavad Mowafaghian Centre for Brain Health, University of British Columbia, Vancouver, British Columbia, Canada

^5^Centre de recherche CHU Sainte Justine, Department of Psychiatry and Addictology, University of Montreal, Montreal, Québec, Canada

^6^Department of Child Health and Development, Norwegian Institute of Public Health, Oslo, Norway

^7^Department of Neurosciences 'Rita Levi Montalcini', University of Turin, Turin, Italy

^8^Eating Disorders Center for Treatment and Research, University of Turin, Turin, Italy

^9^PROMENTA Research Center, Department of Psychology, University of Oslo, Oslo, Norway

^10^Division of Mental Health and Substance Abuse, Diakonhjemmet Hospital, Oslo, Norway

^11^Institute of Psychiatry, Psychology and Neuroscience, King’s College London, London, United Kingdom

^12^Eating Disorders Unit, Department of Psychological Medicine, King’s College London, London, United Kingdom

^13^Department of Psychiatry, University of California San Diego, La Jolla, California, USA

^14^Eating Disorders Center for Treatment and Research, University of California San Diego, La Jolla, California, USA

^15^Bielefeld University, Medical School and University Medical Center OWL, Protestant Hospital of the Bethel Foundation, Department of Child and Adolescent Psychiatry, Bielefeld, Germany

^16^Department of Child and Adolescent Psychiatry, University Clinic Erlangen, Erlangen, Germany

^17^Institute of Experimental and Clinical Pharmacology and Toxicology, Emil Fischer Center, University of Erlangen-Nuremberg, Erlangen, Germany

^18^Department of Neuroradiology, University of Erlangen-Nuremberg, Erlangen, Germany

^19^FAU NeW - Research Center for New Bioactive Compounds, Friedrich-Alexander-Universität Erlangen-Nürnberg, Erlangen, Germany

^20^Department of Neuroradiology, University of Erlangen-Nuremberg, Erlangen, Germany

^21^Centre for Psychosocial Medicine, Department of General Internal Medicine and Psychosomatics, University Hospital Heidelberg, Heidelberg, Germany

^22^Department of Neuroscience, University of Padova, Padova, Italy

^23^Department of Psychiatry and Behavioral Sciences, University of Texas Health Science Center, Houston, Texas USA

^24^Centre for Addiction and Mental Health, Toronto, Ontario, Canada

^25^UMC Utrecht Brain Center, Utrecht University, Utrecht, the Netherlands

^26^Altrecht Eating Disorders Rintveld, Altrecht Mental Health Institute, Zeist, the Netherlands; Faculty of Social Sciences, Utrecht University, Utrecht, the Netherlands

^27^Division of Human Nutrition and Health, Wageningen University, Wageningen, the Netherlands

^28^Imaging Genetics Center, Stevens Institute for Neuroimaging and Informatics, Keck USC School of Medicine, Marina del Rey, California, USA

^29^Department of Psychiatry, Icahn School of Medicine at Mount Sinai, New York, New York, USA

**Table of contents**

**A Methods**

A.1 Ethical approval and previous publications

A.2 Study criteria

A.3 Assessment of subtype

A.4 FreeSurfer preprocessing

A.5 Harmonization of site-effects

A.6 Normative modelling

A.7 Classification pipelines

A.8 Hyperparameter optimization

A.9 Performance estimation

A.10 Permutation tests for model performance

A.11 Leave-Sites-Out cross-validation

A.12 Permutation tests for confounding variables

A.13 Feature importance and reliability

**B Results**

B.1 Heterogeneity of z-scores in AN

B.2 Proportions of participants with extreme z-scores in AN-R and AN-BP

B.3 Participant-level load of extreme structural deviations in AN vs. HC and AN-BP vs. AN-R

**References**

**Tables**

Table A. Descriptive statistics per site (AN vs. HC analyses)

Table B. Descriptive statistics per site (AN-R vs. AN-BP analyses)

Table C. Imaging specification per site

Table D. Optimal hyperparameters found with grid search

Table E. Comparison of proportions of infranormal/supranormal z-scores (AN-BP vs. AN-R)

Table F. Participant-level load of extreme z-scores (group comparisons)

Table G. Deviance explained by ML risk scores and confounds (AN vs. HC)

**Figures**

Figure A. Proportion of participants with AN and HC per site

Figure B. Age and BMI distributions (AN vs. HC) per site

Figure C. Proportion of AN-R and AN-BP within AN per site

Figure D. Age and BMI distributions (AN-R vs. AN-BP) per site

Figure E. Univariate CT comparisons (AN vs. HC)

Figure F. Univariate SA + subcortical volume comparisons (AN vs. HC)

Figure G. Normative-model percentiles: subcortical volumes (HC vs. AN)

Figure H. Normative-model percentiles: subcortical volumes (AN-R vs. AN-BP)

Figure I. Overlap of regions with elevated extreme z-score proportions across subtypes

Figure J. SVM performance (AN vs. HC)

Figure K. SVM performance (AN-BP vs. AN-R)

Figure L. Neural network performance (AN vs. HC)

Figure M. Neural network performance (AN-BP vs. AN-R)

# A Methods

**A.1 Ethical approval and previous publications**

All participating cohorts obtained approval from local institutional review boards and ethics

committees, and all study participants provided written informed consent. We report in the following the approvals for each site.

**Denver:** The study was approved by the Colorado Multiple Institutional Review Board (protocol number: 07-0816). We obtained written assent from the participant and consent from the legal guardian prior to conducting any of the study procedures.

**Dresden:** The study received approval from the Ethics Commission at Dresden Technical University (protocols: EK 39022012, EK 536122015, EK 536122015), and all participants or their legal guardians provided written informed consent.

**Erlangen:** The Ethics Committee of the University Hospital of Erlangen gave approval for the study

and it was conducted in accordance with the Declaration of Helsinki (220_15B).

**Heidelberg:** The medical ethics committee of the Medical Faculty Heidelberg at the Ruprecht-Karls-University in Heidelberg, Germany, approved this study, and written informed consent was obtained from all participants (protocol numbers S-373/2014, S-592/2015, S-125/201).

**London (BRCACE and PREDICTA):** The study received ethical approval from the London—City Road and Hampstead Research Ethics Committee and the King's College London Psychiatry, Nursing and Midwifery Research Ethics Subcommittee (References: 15/LO/0196 and HR-15/16-2836).

**Oslo:** This study is approved by the Norwegian Regional Committee for Medical and Health Research Ethics. Written informed consent was obtained from all participants (2015/793).

**Padova:** Ethical permission was obtained from the ethics committee of the Padova Hospital. After completely describing the study to the subjects, written informed consent was obtained (ID 1598P).

**San Diego (AdolAN and sMRI):** The study was conducted according to the IRB regulations of the University of California, San Diego Human Research Protections Program (180883). The AdolAN and sMRI cohorts from this site were treated as different (particularly during ComBat-GAM harmonization and LSsO) because two different scanners were used (GE 3T Signa Excite vs. GE 3T MR750).

**Torino:** The study was approved by the local Ethics Committee [Comitato Etico Interaziendale A.O.U. Città della Salute e della Scienza di Torino—A.O. Ordine Mauriziano—A.S.L. Città di Torino; approval #12042010].

**Toronto:** The study was approved by the Centre for Addiction and Mental Health Research Ethics Board (protocol number: CAMH REB #144/2013). All participants gave written informed consent.

**Utrecht (WOFAN and URGE):** This study was approved by the Institutional Review Board of the University Medical Center (Utrecht). Participants provided written informed consent (NL28258.041.09, NL45093.041.13, Dutch trial registry nr NL4199 (NTR4351)). The WOFAN and URGE cohorts from this site were treated as being one (particularly during ComBat-GAM harmonization and LSsO) because the same scanner and scanning protocol were used.

The current manuscript is a coordinated mega-analysis of datasets, which have mostly been published on (e.g., [1]). Our work is substantively different from the previously published work in that: i) imaging analysis, quality control and statistical analysis methods across datasets were designed a priori using harmonized protocols and did not rely on published findings, hence reducing the risk of publication bias; ii) our work included an unprecedented number of international research sites (570 patients and 739 healthy controls in 13 cohorts from 11 sites worldwide) generating more generalizable and rigorous findings compared to a single large study; iii) we conducted analyses that go beyond case-control comparisons (or comparisons between AN subtypes) and include deviations from the normative reference based on the CentileBrain normative model [2], and machine learning classification.

**A.2 Study criteria**

Inclusion and exclusion criteria were as in Walton et al. [3], but participants without AN subtype information were excluded from the analyses comparing AN-R vs AN-BP. Exclusion criteria for participants in both the AN and HC group were: current comorbid severe psychiatric disorders (such as schizophrenia/schizoaffective, bipolar disorder, substance dependence), current severe neurological disorders, significant current or chronic medical illness that can explain most of the weight loss, significant lifetime neurological illness/ accidents that may have affected the brain, preterm birth <30 weeks, and/or developmental disorders.

We subcategorized participants with AN according to subtype (restricting or binge-purge), when this information was available. Participants in both subgroups (AN-R and AN-BP) were at different stages of weight recovery at the time of their MRI scan: some participants had just started a weight restoration treatment and were therefore acutely underweight, while others had already been in treatment for some time and/or gained some weight). However, no participant had yet reached their target weights. Participants were included in the AN group if their BMI was <18.5 (adults) or below the 10^th^ age-adjusted percentile (adolescents), as originally proposed by Hebebrand et al. [4]. If BMI was between 18.5–19.5 (adults) or between the 10^th^ and 25^th^ age-adjusted percentile, participants were still included if they were engaged in regular therapy for AN, had no regular menses (unless on birth control medication), and continued to show significant eating disorder symptoms (6/570). Participants with obesity (BMI>30) were excluded. Inclusion and exclusion criteria were part of our a priori defined study design. BMI was estimated as the body mass divided by the square of the body height and expressed in units of kg/m². When using English measurements (e.g., pounds (lb) and inches (in) the result can be transformed using a conversion factor of 703).

Criteria were discussed and defined during the regular telephone conferences within the ENIGMA (Enhancing NeuroImaging Genetics through Meta-Analysis) Eating Disorders (ED) Working Group which includes ED Specialists and Clinician-Scientists from around the world. The criteria were posted on the ENIGMA website in 2018 (http://enigma.ini.usc.edu/wp-content/uploads/2019/02/grouping_guidelines_ENIGMA_AN_ms_201812.pdf).

**A.3 Assessment of subtype**

**Denver:** All participants were assessed by a doctoral-level interviewer with the Structured Clinical Interview for DSM-5 Disorders (SCID; [5]) for general psychiatric and eating disorder specific diagnoses including anorexia nervosa subtype.

**Dresden:** Subtype was assessed using the expert form of the Structured Interview for Anorexia and Bulimia Nervosa (SIAB-EX) [37]. Interviews were adapted to DSM-5 criteria [6] and carried out by clinically experienced and trained research assistants under the supervision of the attending child and adolescent psychiatrist.

**Erlangen:** Diagnosis and subtype were assessed according to ICD-10 criteria by an experienced psychiatrist or psychologist using the "Diagnostic System for Mental Disorders for Children and Adolescents (DISYPS-II)" [7] in adolescents and the "Structured Diagnostic Interview for Mental Disorders (DIPS)" [8] in adults.

**Heidelberg:**  Each participant was screened for medical and psychiatric disorders using the “Structured Clinical Interview for Diagnostic and Statistical Manual of Mental Disorders, 4th edition” (SCID; [9]). AN-subtype was subsequently determined based on DSM-5 criteria [6].

**London:** Subtype was assessed using the research version of the “Structured Interview for Anorexia and Bulimia Nervosa” (SCID) for DSM IV [10]. Interviews were carried out by clinically experienced and trained research assistants under the supervision of an attending psychiatrist.

**Oslo:** In this site subtype was not assessed. Therefore, participants from this site were excluded for the analyses on AN-subtype.

**Padua:** Subtype was assessed by means of the eating disorders section of the “Structured Clinical Interview for DSM-IV (APA)” adapted to DSM-5 criteria.

**San Diego (AdolAN and sMRI):** Axis I diagnoses were made by a child and adolescent psychiatrist with expertise in adolescents with an eating disorder. Assessments included the “Mini International Neuropsychiatric Interview for Children and Adolescents” (MINI-KID; [11]) and a modified Module H (eating disorder diagnosis) from the DSM-IV that included additional questions to further define eating disorder characteristics.

**Torino:** Subtype was assessed with clinical interview by experienced psychiatrists based on DSM-IV-TR criteria.

**Toronto:** Subtype was assessed using a comprehensive clinical battery, including a semi-structured clinical interview; relevant modules from the Structured Clinical Interview for Diagnosis, Research Version (SCID-I/P; [5]); and the Eating Disorder Examination Questionnaire (EDE-Q 6.0; [12]). Assessments were performed by a clinically experienced graduate trainee under the supervision of two senior psychiatrists, Drs. Allan Kaplan and Aristotle Voineskos.

**Utrecht (WOFAN and URGE):** In both cohorts, patients with AN were recruited at Altrecht Eating Disorders Rintveld in Zeist, the Netherlands. Rintveld is a nationwide tertiary referral center for patients with eating disorders that offers diagnostic assessments, consultation, and personalized treatment for in- and outpatients. The AN diagnoses (including subtype) were established by eating disorder experts (psychiatrists or clinical psychologists) with ample experience in the assessment and treatment of eating disorders, and confirmed by the Eating Disorder Examination [13].

**A.4 FreeSurfer preprocessing**

The ENIGMA protocol for quality assurance was performed at each site prior to analysis, and included visual checks of the cortical segmentations and region-by-region removal of values for segmentations found to be incorrect (http://enigma.usc.edu/protocols/imaging-protocols). Histograms of all regions’ values for each site/ sample were also produced for visual inspection. All cohorts carried out the same processing steps that are included in the FreeSurfer ‘recon-all’ procedure. Initially, the MRI scans underwent FreeSurfer preprocessing which included motion correction, intensity normalization, Talairach transformation to the MNI305 atlas and skull stripping. Subsequently, subcortical segmentation and cortical surface reconstruction for each hemisphere were performed. This included tessellation of the gray matter-white matter boundary, automated topology correction, and surface deformation following intensity gradients to optimally place the gray-white and gray-cerebrospinal fluid borders at the location where the greatest shift in intensity defines the transition to the other tissue class. Finally, the cortex was parcellated and a neuroanatomical label was assigned to each location on the cortical surface according to the Desikan-Killiany atlas [14]. We refer to the FreeSurfer Documentation for more details (https://surfer.nmr.mgh.harvard.edu/fswiki/recon-all). Working group members had access to the statistical results from each site.

**A.5 Harmonization of site-effects**

We used ComBat-GAM [15] to harmonize brain structural measures across sites. This method is based on ComBat, which was originally proposed to remove batch effects in genomics data [16], and has been applied to neuroimaging data [17]. In short, ComBat removes site-related effects, while preserving variations due to other biologically-relevant covariates in the data (e.g., age). While ComBat only preserves linear dependence of covariates, ComBat-GAM extends the original ComBat method by incorporating Generalized Additive Models (GAMs) to model non-linear relationships between measure of brain structure and potential confounding factors. Specifically, following Pomponio et al. [15], we used GAMs to model age dependence, and a linear model for BMI. Subtype and diagnosis dependences (if present) were also preserved. For SA and subcortical volumes, also the linear dependence in estimated intracranial volume (ICV) was preserved.

**A.6 Normative Modelling**

To compute individual deviations relative to a normative reference population we used the CentileBrain public models [2, 18, 19]. These models use multivariate fractional polynomial regression to fit sMRI data from 37 407 healthy individuals (53% female and 47% male; aged 3–90 years) from 87 datasets from Europe, Australia, the USA, South Africa, and east Asia. Following extensive benchmarking, the models are based on multivariate fractional polynomial regression, emerged optimising non-linear polynomials for age and linear effects of global metrics as covariates.

We aimed to identify the regional metrics in which a significantly high percentage of participants with AN exhibited supranormal or infranormal z-scores. To this end, we calculated the probability (p-value) of observing the proportion of extreme z-scores in each region, assuming an expected proportion of 2.5% (based on our threshold of z>1.96 for supranormal z-scores and z<-1.96 for infranormal z-scores). The resulting p-values were corrected for multiple comparisons using the false discovery rate (FDR) procedure [20]. The smallest proportions yielding an FDR-corrected p-value *q*<.05 were used as significance thresholds—i.e., any proportion above these thresholds was considered significantly elevated.

The resulting thresholds were as follows:

(a) supranormal z-scores for cortical thickness (CT): 4.04%,

(b) infranormal z-scores for CT: 4.21%,

(c) supranormal z-scores for surface area (SA): 4.21%, and

(d) infranormal z-scores for subcortical volume (SV): 4.39%.

No regions exceeded the significance threshold for infranormal z-scores in SA metrics, nor for supranormal z-scores in SV metrics (Figure B).

To assess heterogeneity in CT, SA, and SV profiles across patients, we computed the standard deviation of z-scores (from the CentileBrain models) across individuals for each metric (separating CT, SA, and SV metrics) and averaged these values to obtain a global heterogeneity index. To evaluate whether this observed heterogeneity exceeded normative expectations, we generated a null distribution by simulating 20000 synthetic datasets drawn from a standard normal distribution (mean=0, SD=1, as in the normative reference) with matched dimensions (number of metrics and number of participants). The one-sided p-value was calculated as the proportion of null samples with a mean standard deviation equal to or greater than the observed value.

**A.7 Classification Pipelines**

We trained classifiers to differentiate AN vs. HC and AN-R vs. AN-BP. To avoid information leakage between the train and test set, both preprocessing (see below) and classification steps in the analysis were included in a pipeline (using the scikit-learn package), that underwent a cross-validation procedure to estimate hyperparameters and prediction performance. The analysis pipeline was similar to the one used in the pilot study by Arold et al. [21], but included a preliminary preprocessing step to harmonize data from different sites using ComBat-GAM as in the univariate analyses [15]. Subsequently, as in Arold et al. [21], we subtracted linear covariate dependences from brain structural measures [22], i.e., age (for all measures and both the AN vs. HC and the AN-R vs. AN-BP classifiers), BMI (for all measures but only for the AN-R vs. AN-BP classifier), and ICV (for both AN vs. HC and AN-R vs. AN-BP classifiers, but just for regional surface area measures and subcortical volumes). We chose to estimate the effect of these covariates only on the largest group (HC and AN-R) to avoid subtracting disease or AN-subtype related effects (Dukart et al. 2011). This adjustment aimed to enforce the classifier to take decisions based on brain structural measures themselves, rather than imbalances between groups in covariates with an effect on brain structure. Then, we applied principal component analysis (PCA) to the residuals of the harmonized regional morphometry measures for dimensionality reduction. Finally, the preprocessed measures derived from ComBat-GAM, linear regression, and PCA were utilized as input features for a linear Support Vector Machine classifier (SVM).

To assess whether non-linear patterns of brain structural alterations were associated with AN, we tested whether using a non-linear classifier could substantially improve the classification performance. Specifically, the SVM was substituted in the classification pipeline by a neural network with one hidden layer. We explored a hidden layer with sizes of two, five, six or ten neurons, different activation functions (sigmoid, ReLU), and L2 regularization values 1, 10, or 20. Considerably more neurons, additional hidden layers, or weaker regularization increase the model complexity too much and thus produced strong overfitting judging from learning curves. While these comparably low numbers of hidden neurons only produce a weak non-linear extension, it was shown that any decision boundary can be approximated by a continuous feed-forward neural network with only a single internal hidden layer [23]. We also tested other non-linear classification algorithms (radial-basis-function kernel SVM, Random Forest) but found them all to not exceed significantly the linear SVM performance. Thus, they were not considered further.

**A.8 Hyperparameter Optimization**

Our classification pipelines had free hyper-parameters. For example, in our linear classification pipeline the number of PCA components (in percent of explained variance) and the SVM regularization constant $C$ were free hyperparameters. Therefore, our model selection procedure included both optimization of the model parameters (which we refer to as training) and of the hyperparameters.

To optimize the hyperparameters we performed grid search with ten-fold cross-validation, stratified to maintain the proportion of groups (AN vs. HC and AN-BP vs. AN-R) equal across folds. We used PR-AUC as optimization metric. Compared to the more traditional metric accuracy, this metric captures the trade-off between positive-predictive-value/precision and sensitivity/recall in an unbalanced dataset.

In cross-validation, the dataset is partitioned into the given number of folds to repeatedly aggregate training/validation splits of the data. In each split, a different fold is held out and the model is trained on the remaining folds (training set). Subsequently, the held-out fold (validation set) is used to assess the performance of the trained model. In the context of grid search, this procedure is repeated for each combination of the hyperparameters. The best hyperparameters were selected as those optimizing performance on the validation set, measured as the average across splits. For further details on the grid values and results, see Table D.

**A.9 Performance estimation**

To estimate the performance of the classifiers obtained using the above-mentioned model selection procedure (optimization of the model parameters and hyperparameters) we applied nested cross-validation. In nested cross-validation, the above-described model selection via cross-validation was embedded in an outer ten-fold and stratified cross-validation loop. As a result, in each split the entire dataset is divided in three: (i) the test set (one outer fold), used to assess the performance of the model selection procedure, (ii) the validation set (one inner fold), used to optimize the hyperparameters, and (iii) the training set (remaining inner folds), used to optimize the model parameters. Therefore, the final measure of model performance was obtained from the average test performance (Figures J-M). The latter is the average over all outer cross-validation splits, where the model with optimal hyperparameters is retrained on the outer training set and its performance is evaluated on the so far unseen test set.

The difference between the test and validation performance (commonly called generalization gap) can inform whether the model obtained (with optimal parameters and hyperparameters) will generalize well to unseen data. This is the case if the generalization gap is small. Conversely, a large generalization gap is an indication that the model is overfitting the training and validation data.

**A.10 Permutation tests for model performance**

To test whether the trained classifiers performed significantly better than chance, we employed permutation tests [24, 25] as described by [26].

To construct the null distribution of performances for our model fitting procedure we first determined the optimal hyperparameters on the whole dataset using cross-validation as described above. For each permutation of the labels (i.e., AN vs. HC, or AN-BP vs. AN-R) we computed AUC as an average over cross-validation splits (stratified, ten-fold). This procedure was repeated 10000 times. Finally, the probability to obtain a performance equal or higher than the one estimated with nested cross-validation was computed, based on the empirical null distribution of permuted AUCs obtained.

**A.11 Leave-Sites-Out cross validation**

Leave-sites-out cross-validation (LSsO) is a model validation technique primarily used in spatial and environmental studies where the data points are geographically or environmentally clustered. Unlike standard cross-validation methods that randomly partition the data, LSsO involves systematically leaving out entire clusters or "sites" of data during model training. This approach ensures that the model's performance is evaluated on truly independent data, mimicking real-world scenarios where predictions are made for new locations or conditions not seen during training. By validating the model in this manner, LSsO helps to assess its generalizability and robustness, particularly in the presence of spatial autocorrelation or site-specific variations. This approach was employed in another ENIGMA study employing machine learning to classify participants with bipolar disorder from healthy controls [27]. While in that study data from one site were completely excluded from the training partition, here we allowed more than one site to be excluded from the training partition in the cross-validation loop. This allowed us to optimize folds in such a way that the proportion of participants across groups and sizes of partitions were more similar across folds. Importantly, since in this validation technique it is not assumed that data from all sites are available during training, harmonization (and subtraction) of sites effects (e.g., using ComBat-GAM) cannot be performed. Due to significant variations in size and class ratios across sites, we opted for a three-fold stratified cross-validation approach to mitigate the impact of these imbalances.

**A.12 Permutation tests for confounding variables**

Since classifiers are highly sensitive to any kind of exploitable group differences in the data, we also assessed to which extent the information used for classification originated from confounding variables as opposed to sMRI measures, by applying the post-hoc method proposed by Dinga et al. [28]. In short, the method aims to establish, after the machine learning model is fitted, the fraction of deviance explained by: (i) exclusively machine learning predictions (ii) exclusively confounds, and (iii) both machine learning predictions and confounds. Deviance is a goodness-of-fit statistic analogous to explained variance but for categorical variables, such as the labels that indicate group membership (AN vs. HC, or AN-R vs. AN-BP). The machine learning-based risk scores of the classifier trained on the whole dataset were taken as the machine learning predictions. For each data point, they corresponded to the signed distance from the separating hyperplane for SVMs, or the probability output for neural networks. As for performance estimation, permutation tests were used to assess whether fractions of deviance explained exclusively by the machine learning-based risk score were significantly bigger than zero [29]. Importantly, in this case only sMRI features were permuted relative to the group variable but confounds remained unpermuted [28, 30]. As above, for each permutation we computed the fractions of deviance explained as an average over cross-validation splits (stratified, ten-fold).

**A.13 Feature importance and reliability**

In order to evaluate the extent to which each feature impacted the classifier’s decision, we utilized the method of feature importance estimation as outlined in Haufe et al. [31]. For our specific scenario of binary classification with a linear model, this involved calculating the Pearson correlation coefficients between the classifier’s machine learning-based risk score and each feature. For features with positive importance, a higher value indicates a higher likelihood of being classified in the AN group (for the AN vs. HC classification) or in the AN-BP group (for the AN-BP vs. AN-R classification). Conversely, for features with negative importance, a lower value indicates a higher likelihood of being classified in the AN or AN-BP groups. To determine if the feature importances were statistically significant, we computed the Bonferroni-corrected p-values of the Pearson correlation coefficients.

**B Results**

**B.1 Heterogeneity of z-scores in AN**

Group-level heterogeneity indices were elevated across all structural metrics. The mean standard deviation of z-scores across regions was 1.0482 for CT, 1.0131 for SA, and 1.0917 for SV. These values were significantly higher than expected under normative assumptions, as determined by bootstrapping against a null distribution generated from standard normal data (null mean=0.9996 for all metrics; p<.001 for CT and SV, p=<.001 for SA).

**B.2 Proportions of participants with extreme z-scores in AN-R and AN-BP**

In individuals with AN-R, 32/68 of CT metrics showed a significantly higher-than-expected proportion of participants with supranormal z-scores based on the normative reference, while 21/68 showed a significantly higher-than-expected proportion of participants with infranormal z-scores. The corresponding percentages for SA were 17/68 for supranormal values, and 0/68 for infranormal z-scores. The corresponding percentages for SV were 0/14 for supranormal z-scores and 13/14 for infranormal z-scores.

In individuals with AN-BP, 20/68 of CT metrics showed a significantly higher-than-expected proportion of participants with supranormal z-scores based on the normative reference, while 10/68 showed a significantly higher-than-expected proportion of participants with infranormal z-scores. The corresponding percentages for SA were 1/68 for supranormal values, and 0/68 for infranormal z-scores. The corresponding percentages for SV were 0/68 for supranormal z-scores and 10/14 for infranormal z-scores. Overlaps and non-significant differences between subtypes in the metrics where higher proportions of infranormal and supranormal z-scores were observed are reported in Section B.2 and displayed in Figure I.

**B.3 Participant-Level Load of Extreme Structural Deviations in AN vs. HC and AN-BP vs. AN-R**

Taking a different approach compared to the analysis presented in the main text, where we identified the regional metrics where a significantly high number of participants had an extreme z-score, we computed here the percentage of participants in each group that had a significantly high number of extreme z-scores across all metrics of CT, SA, and SV, separately. The probability for the occurrence of an infra-/supranormal z-score according to our definition (z<-1.96, z>1.96) in each metric was 2.5%. We computed the probabilities for the occurrences of N or more infra-/supranormal z-scores across (i) all regional metrics of CT according to the Desikan-Killiany atlas (n=68; Desikan et al. 2006), (ii) all metrics of SA (n=68; Desikan et al. 2006), and (iii) all subcortical volumes (n=14). We found that this probability was smaller than 5% for n>4 for CT or SA metrics and n>1 for SV metrics. Therefore, we defined n>4 and n>1 to be significantly high numbers of infra-/supranormal z-scores (p<0.05) for measures of CT or SA, and SV, respectively. The percentages of participants with a significantly high number of extreme z-scores in AN and HC are reported in Table E. For CT, the proportion of participants with an abnormally high number of supranormal z-scores was increased in AN compared to HC (χ^2=5.72, p=0.017). For SV, the proportion of participants with an abnormally high number of supranormal z-scores (χ^2=9.57, p=0.002) was reduced in AN compared to HC and the proportion of participants with an abnormally high number of infranormal z-scores (χ^2=55.24, p<.001) was increased in AN compared to HC. All other differences were not significant. The percentages of participants with a significantly high number of extreme z-scores in AN-BP and AN-R are reported in Table E. None of the group differences were significant (p>.16).

**References**

1. Bahnsen K, Bernardoni F, King JA, Geisler D, Weidner K, Roessner V, et al. Dynamic Structural Brain Changes in Anorexia Nervosa: A Replication Study, Mega-analysis, and Virtual Histology Approach. J Am Acad Child Adolesc Psychiatry. 2022:S0890856722001836.

2. Ge R, Yu Y, Qi YX, Fan Y, Chen S, Gao C, et al. Normative modelling of brain morphometry across the lifespan with CentileBrain: algorithm benchmarking and model optimisation. Lancet Digit Health. 2024;6:e211–e221.

3. Walton E, Bernardoni F, Batury V-L, Bahnsen K, Larivière S, Abbate-Daga G, et al. Brain Structure in Acutely Underweight and Partially Weight-Restored Individuals With Anorexia Nervosa: A Coordinated Analysis by the ENIGMA Eating Disorders Working Group. Biol Psychiatry. 2022;92:730–738.

4. Hebebrand J, Casper R, Treasure J, Schweiger U. The need to revise the diagnostic criteria for anorexia nervosa. J Neural Transm Vienna Austria 1996. 2004;111:827–840.

5. Spitzer RL. The Structured Clinical Interview for DSM-III-R (SCID): I: History, Rationale, and Description. Arch Gen Psychiatry. 1992;49:624.

6. American Psychiatric Association. Diagnostisches und Statistisches Manual Psychischer Störungen DSM-5®. 2nd edn. Hogrefe; 2018.

7. Döpfner M, Görtz-Dorten A, Lehmkuhl G, Breuer D, Goletz H. DISYPS-II: Diagnostik-System für psychische Störungen nach ICD-10 und DSM-IV für Kinder und Jugendliche – II. Bern: Hans Huber; 2008.

8. Margraf J, Schneider S. Diagnostik psychischer Störungen mit strukturierten Interviews. In: Margraf J, Schneider S, editors. Lehrb. Verhal., Berlin, Heidelberg: Springer Berlin Heidelberg; 2009. p. 339–362.

9. Münster RD. Wittchen, H.-U., Zaudig, M. & Fydrich, T. (1997). SKID Strukturiertes Klinisches Interview für DSM-IV. Achse I und II. Göttingen: Hogrefe, DM 158,-. Hiller, W., Zaudig, M. & Mombour, W. (1997). IDCL Internationale Diagnosen Checklisten für DSM-IV und ICD-10. Göttingen: Hogrefe, DM 198,- bzw. DM 239,-. Z Für Klin Psychol Psychother. 1999;28:68–70.

10. First MGM, Spitzer R, Williams J. User’s guide for the Structured Clinical Interview for Diagnosis for DSM-IVTR axis I disorders: Research version. New York: Biometrics Research; 2001.

11. Sheehan DV, Sheehan KH, Shytle RD, Janavs J, Bannon Y, Rogers JE, et al. Reliability and validity of the Mini International Neuropsychiatric Interview for Children and Adolescents (MINI-KID). J Clin Psychiatry. 2010;71:313–326.

12. Fairburn CG, Beglin SJ. Eating Disorder Examination Questionnaire. 2011.

13. Fairburn CG, Cooper Z. The Eating Disorder Examination (12th ed.). Binge Eat. Nat. Assess. Treat., New York: Guilford Press; 1993. p. 317–360.

14. Desikan RS, Ségonne F, Fischl B, Quinn BT, Dickerson BC, Blacker D, et al. An automated labeling system for subdividing the human cerebral cortex on MRI scans into gyral based regions of interest. NeuroImage. 2006;31:968–980.

15. Pomponio R, Erus G, Habes M, Doshi J, Srinivasan D, Mamourian E, et al. Harmonization of large MRI datasets for the analysis of brain imaging patterns throughout the lifespan. NeuroImage. 2020;208:116450.

16. Johnson WE, Li C, Rabinovic A. Adjusting batch effects in microarray expression data using empirical Bayes methods. Biostatistics. 2007;8:118–127.

17. Fortin J-P, Cullen N, Sheline YI, Taylor WD, Aselcioglu I, Cook PA, et al. Harmonization of cortical thickness measurements across scanners and sites. NeuroImage. 2018;167:104–120.

18. Frangou S, Modabbernia A, Williams SCR, Papachristou E, Doucet GE, Agartz I, et al. Cortical thickness across the lifespan: Data from 17,075 healthy individuals aged 3–90 years. Hum Brain Mapp. 2022;43:431–451.

19. Dima D, Modabbernia A, Papachristou E, Doucet GE, Agartz I, Aghajani M, et al. Subcortical volumes across the lifespan: Data from 18,605 healthy individuals aged 3–90 years. Hum Brain Mapp. 2022;43:452–469.

20. Benjamini Y, Hochberg Y. Controlling the False Discovery Rate: A Practical and Powerful Approach to Multiple Testing. J R Stat Soc Ser B Methodol. 1995;57:289–300.

21. Arold D, Bernardoni F, Geisler D, Doose A, Uen V, Boehm I, et al. Predicting long-term outcome in anorexia nervosa: a machine learning analysis of brain structure at different stages of weight recovery. Psychol Med. 2023:1–10.

22. Snoek L, Miletić S, Scholte HS. How to control for confounds in decoding analyses of neuroimaging data. NeuroImage. 2019;184:741–760.

23. Cybenko G. Approximation by superpositions of a sigmoidal function. Math Control Signals Syst. 1989;2:303–314.

24. Noirhomme Q, Lesenfants D, Gomez F, Soddu A, Schrouff J, Garraux G, et al. Biased binomial assessment of cross-validated estimation of classification accuracies illustrated in diagnosis predictions. NeuroImage Clin. 2014;4:687–694.

25. Golland P, Fischl B. Permutation Tests for Classification: Towards Statistical Significance in Image-Based Studies. In: Taylor C, Noble JA, editors. Inf. Process. Med. Imaging, vol. 2732, Berlin, Heidelberg: Springer Berlin Heidelberg; 2003. p. 330–341.

26. Ojala M, Garriga GC. Permutation tests for studying classifier performance. J Mach Learn Res. 2010;11.

27. Nunes A, Schnack HG, Ching CRK, Agartz I, Akudjedu TN, Alda M, et al. Using structural MRI to identify bipolar disorders – 13 site machine learning study in 3020 individuals from the ENIGMA Bipolar Disorders Working Group. Mol Psychiatry. 2018. 31 August 2018. https://doi.org/10.1038/s41380-018-0228-9.

28. Dinga R, Schmaal L, Penninx BWJH, Veltman DJ, Marquand AF. Controlling for effects of confounding variables on machine learning predictions. Bioinformatics; 2020.

29. Ojala M, Garriga GC. Permutation Tests for Studying Classifier Performance. 2009 Ninth IEEE Int. Conf. Data Min., Miami Beach, FL, USA: IEEE; 2009. p. 908–913.

30. Höfling H, Tibshirani R. A study of pre-validation. Ann Appl Stat. 2008;2.

31. Haufe S, Meinecke F, Görgen K, Dähne S, Haynes J-D, Blankertz B, et al. On the interpretation of weight vectors of linear models in multivariate neuroimaging. NeuroImage. 2014;87:96–110.

**Tables**

**Table A. Descriptive statistics per site, anorexia nervosa (AN) vs. healthy controls (HC) analyses.** N=sample size; SD=standard deviation; BMI=body mass index in kg/m²; DURILL=duration of illness in years; AO=age of onset in years.

| **SITE** | **HC** |  |  |  |  |
| --- | --- | --- | --- | --- | --- |
|  | **N** | **age (mean)** | **age (std)** | **BMI (mean)** | **BMI (std)** |
|  |  |  |  |  |  |
| Oslo | 25 | 17.88 | 2.35 | 21.73 | 2.84 |
| BRCACE | 35 | 26.86 | 5.28 | 21.96 | 1.95 |
| Denver | 136 | 21.43 | 6.44 | 21.18 | 2.02 |
| Dresden | 301 | 18.91 | 4.40 | 21.05 | 2.20 |
| Erlangen | 28 | 20.90 | 5.57 | 21.77 | 2.41 |
| Heidelberg | 59 | 23.67 | 3.75 | 21.32 | 1.50 |
| PREDICTA | 21 | 25.71 | 9.07 | 22.30 | 2.46 |
| Padova | 38 | 25.01 | 6.72 | 21.67 | 2.94 |
| Torino | 17 | 22.24 | 2.73 | 21.46 | 2.34 |
| UCSD_AdolAN | 10 | 15.28 | 1.79 | 20.81 | 2.02 |
| UCSD_sMRI | 13 | 15.70 | 1.80 | 21.33 | 2.32 |
| Toronto | 24 | 25.04 | 5.68 | 22.26 | 2.18 |
| Utrecht | 32 | 24.81 | 4.10 | 21.56 | 1.95 |

| **SITE** | **AN** |  |  |  |  |  |  |  |  |  |  |
| --- | --- | --- | --- | --- | --- | --- | --- | --- | --- | --- | --- |
|  | **N** | **age (mean)** | **age (std)** | **BMI (mean)** | **BMI (std)** | **DURILL (mean)** | **DURILL (std)** | **AO (mean)** | **AO (std)** | **proportion of AN-BP** | **proportion of AN-R** |
|  |  |  |  |  |  |  |  |  |  |  |  |
| Oslo | 8 | 19.00 | 1.77 | 15.52 | 1.35 | nan | nan | nan | nan | 0.00 | 0.00 |
| BRCACE | 28 | 26.21 | 7.60 | 15.44 | 1.19 | 9.16 | 8.29 | 16.92 | 4.12 | 0.21 | 0.75 |
| Denver | 114 | 19.47 | 5.60 | 16.35 | 1.26 | 4.97 | 5.23 | 14.53 | 2.88 | 0.22 | 0.78 |
| Dresden | 185 | 16.53 | 3.11 | 14.77 | 1.50 | 1.16 | 1.48 | 15.40 | 2.89 | 0.12 | 0.86 |
| Erlangen | 21 | 22.21 | 7.69 | 14.81 | 1.13 | 5.85 | 7.45 | nan | nan | 0.24 | 0.67 |
| Heidelberg | 62 | 23.34 | 4.65 | 15.24 | 1.40 | 5.24 | 4.33 | 18.13 | 4.22 | 0.31 | 0.69 |
| PREDICTA | 23 | 26.96 | 8.03 | 15.65 | 1.25 | 9.29 | 6.63 | 17.61 | 6.75 | 0.35 | 0.61 |
| Padova | 37 | 25.81 | 7.04 | 15.90 | 1.47 | 6.08 | 6.20 | 18.49 | 5.05 | 0.65 | 0.35 |
| Torino | 22 | 20.86 | 4.84 | 15.91 | 0.84 | 0.95 | 0.63 | nan | nan | 0.18 | 0.82 |
| UCSD_AdolAN | 9 | 17.03 | 1.93 | 17.49 | 0.83 | 2.77 | 2.29 | 14.00 | 1.63 | 0.00 | 0.78 |
| UCSD_sMRI | 13 | 15.49 | 1.64 | 17.78 | 1.03 | 1.43 | 0.96 | 14.00 | 1.73 | 0.08 | 0.92 |
| Toronto | 20 | 28.20 | 7.42 | 15.64 | 1.15 | 12.60 | 7.84 | nan | nan | 0.60 | 0.40 |
| Utrecht | 28 | 24.18 | 5.70 | 14.99 | 1.57 | 7.54 | 4.78 | 15.33 | 2.77 | 0.32 | 0.64 |

**Table B. Descriptive statistics per site, anorexia nervosa of restricting subtype (AN-R) vs. anorexia nervosa of binge-eating/purging subtype** **(AN-BP) analyses.** N=sample size; SD=standard deviation; BMI=body mass index in kg/m²; DURILL=duration of illness in years; AO=age of onset in years.

|  | **subtype AN-R** |  |  |  |  |  |  |  |  |
| --- | --- | --- | --- | --- | --- | --- | --- | --- | --- |
|  | **N** | **age (mean)** | **age (std)** | **BMI (mean)** | **BMI (std)** | **DURILL (mean)** | **DURILL (std)** | **AO (mean)** | **AO (std)** |
| **SITE** |  |  |  |  |  |  |  |  |  |
| **BRCACE** | 21 | 26.14 | 8.36 | 15.45 | 1.29 | 9.65 | 8.78 | 16.65 | 4.30 |
| **Denver** | 89 | 18.76 | 4.88 | 16.12 | 1.15 | 4.29 | 4.60 | 14.49 | 2.84 |
| **Dresden** | 160 | 16.31 | 2.95 | 14.70 | 1.52 | 1.08 | 1.36 | 15.25 | 2.67 |
| **Erlangen** | 14 | 20.08 | 6.61 | 14.55 | 1.04 | 5.07 | 7.35 | nan | nan |
| **Heidelberg** | 43 | 23.12 | 4.66 | 15.10 | 1.40 | 4.43 | 3.93 | 18.79 | 4.44 |
| **PREDICTA** | 14 | 25.71 | 7.27 | 15.21 | 1.29 | 9.41 | 7.79 | 16.21 | 4.00 |
| **Padova** | 13 | 25.48 | 6.86 | 14.93 | 1.76 | 3.35 | 3.84 | 20.77 | 6.47 |
| **Torino** | 18 | 20.83 | 5.33 | 15.95 | 0.76 | 0.94 | 0.64 | nan | nan |
| **UCSD_AdolAN** | 7 | 16.80 | 2.01 | 17.21 | 0.72 | 2.77 | 2.29 | 14.00 | 1.63 |
| **UCSD_sMRI** | 12 | 15.46 | 1.71 | 17.78 | 1.07 | 1.49 | 0.99 | 13.92 | 1.78 |
| **UToronto_ANDT** | 8 | 27.75 | 10.86 | 15.88 | 1.32 | 11.12 | 10.36 | nan | nan |
| **Utrecht** | 18 | 23.11 | 5.71 | 15.20 | 1.50 | 6.33 | 4.07 | 14.88 | 3.00 |

|  | **subtype AN-BP** |  |  |  |  |  |  |  |  |
| --- | --- | --- | --- | --- | --- | --- | --- | --- | --- |
|  | **N** | **age (mean)** | **age (std)** | **BMI (mean)** | **BMI (std)** | **DURILL (mean)** | **DURILL (std)** | **AO (mean)** | **AO (std)** |
| **SITE** |  |  |  |  |  |  |  |  |  |
| **BRCACE** | 6 | 26.50 | 5.68 | 15.60 | 0.79 | 8.25 | 6.85 | 16.75 | 2.50 |
| **Denver** | 25 | 22.00 | 7.17 | 17.16 | 1.33 | 7.36 | 6.58 | 14.64 | 3.07 |
| **Dresden** | 22 | 18.16 | 3.84 | 15.31 | 1.16 | 1.47 | 1.61 | 16.69 | 3.96 |
| **Erlangen** | 5 | 25.62 | 7.31 | 15.80 | 1.05 | 8.83 | 8.28 | nan | nan |
| **Heidelberg** | 19 | 23.85 | 4.71 | 15.56 | 1.38 | 7.02 | 4.73 | 16.68 | 3.37 |
| **PREDICTA** | 8 | 30.12 | 8.98 | 16.30 | 0.89 | 10.00 | 4.14 | 20.12 | 10.12 |
| **Padova** | 24 | 25.99 | 7.28 | 16.42 | 0.98 | 7.57 | 6.78 | 17.25 | 3.69 |
| **Torino** | 4 | 21.00 | 1.83 | 15.72 | 1.30 | 1.00 | 0.71 | nan | nan |
| **UCSD_AdolAN** | 0 | nan | nan | nan | nan | nan | nan | nan | nan |
| **UCSD_sMRI** | 1 | 15.88 | nan | 17.70 | nan | 0.83 | nan | 15.00 | nan |
| **UToronto_ANDT** | 12 | 28.50 | 4.44 | 15.49 | 1.05 | 13.58 | 5.93 | nan | nan |
| **Utrecht** | 9 | 27.00 | 4.90 | 14.64 | 1.79 | 10.56 | 4.95 | 15.67 | 2.52 |

**Table C. Imaging specification per site.** BW=bandwidth, FOV=field of view, FSPGR=Fast Spoiled Gradient-Recalled Echo, MP-RAGE/MPRAGE=Magnetization-Prepared Rapid Gradient-Echo, NEX=number of excitations, R-FSPGR=Radiofrequency-spoiled Fast Spoiled Gradient-Recalled Echo, SENSE=sensitivity encoding (parallel imaging), SPGR=Spoiled Gradient-Recalled Echo, TE=echo time, TFE=Turbo Field Echo, TI=inversion time, TR=repetition time.

| Site | Number Of Scanners | Scanner Vendor | Scanner Type | Field Strength | Imaging Protocols | Voxel size mm | Slice  Orientation | FreeSurfer Version | Operating  System |
| --- | --- | --- | --- | --- | --- | --- | --- | --- | --- |
| Oslo | 2 | General Electric Healthcare/ Siemens | Signa/ Avanto | 1.5T | 3D FSPGR Sequence, TR=2100 ms, TE=6 ms, flip angle=35°, FOV=280 mm/  3D MPR Sequence, TR=1940 ms, TE=3.09 ms, flip angle=15°, FOV=256 mm | 1.09x 1.09x 1.7/ 1x1x1 | coronal/ sagittal | 6,0 | Linux  centos  6x86_64 |
| London BRCACE | 1 | General Electric Healthcare | Signa HDx | 1.5T | MP-Rage Sequence, TR=8.6 ms, TE=3.8 ms, TI=1000 ms, flip angle=8°, slice thickness=1.2 mm, matrix=192x256 mm, 180 slices | 1.2x 0.938x 0.938 | sagittal | 5,3 | Linux  Neurodebian |
| Denver | 2 | General Electric Healthcare/ Siemens | Signa/ Skyra | 3T | Spoiled Gradient Sequence T1-weighted, TR=8 ms, TE=4 ms, TI=450 ms, flip angle=12°, FOV=220 mm, matrix=64x64, slice thickness=1 mm, 172 slices | 1x 1x 1 | sagittal | 5,3 | Linux  Ubuntu |
| Dresden | 1 | Siemens | Magnetom Trio | 3T | MP-Rage Sequence, TR=1900 ms, TE=2.26 ms, flip angle=9°, FOV=256x224 mm², bandwidth of 200Hz/pixel, slice thickness=1 mm, 176 slices, no gap | 1x 1x 1 | sagittal | 5,3 | Linux  Neurodebian |
| Erlangen | 1 | Siemens | Trio | 3T | MP-Rage Sequence, T1-weighted 3D, TR=1900 ms, TE=2.25 ms, flip angle=9°, FOV=256×256×256 mm, slice thickness=1 mm isotropic resolution | 1x 1x 1 | sagittal | 6,0 | Mac  OS X |
| Heidelberg | 1 | Siemens | Magnetom Trio | 3T | MP-Rage Sequence, TR=1.570 ms/TR=1.900 ms, TE=2.63 ms/ TE=2.52 ms, flip angle=9°, FOV=256 mm, 192 slices | 1x 1x 1 | sagittal | 6,0 | Linux  Ubuntu |
| Padova | 1 | Philips | Achieva | 1.5T | 3D T1-weighted, Gradient-Echo Sequence, TR=20 s, TE=3.78 ms, flip angle=20°, FOV=210-220 mm, 160 slices | 1x 0.66x 0.66 | sagittal | 5,3 | Mac Os X  10.11.3 |
| London PREDICTA | 1 | General Electric Healthcare | Signa HDx | 1.5T | MP-Rage Sequence, TR=8.6 ms, TE=3.8, TI=1000 ms, flip angle=8°, matrix=192x256 mm, slice thickness=1.2 mm, 180 slices | 1.2x 0.938x 0.938 | sagittal | 5,3 | Linux  Neurodebian |
| Torino | 1 | Philips | Achieva | 1.5T | 3D T1-weighted Ultrafast 3D Gradient Echo (Philips acronym 3D-TFE, Siemens acronym MP-RAGE), TR=7 ms, TE=3 ms, flip angle=8°, matrix 256x256 mm, FOV=256 mm, pixel BW=241 Hz, slice thickness=1 mm, SENSE=1 x 3, duration 4-5 min, 190 slices | 1x 1x 1 | sagittal | 5,3 | Linux  Ubuntu |
| Utrecht URGE | 1 | Philips | Achieva | 3T | 3D TFE Sequence, TR=8.4 ms, TE=3.8 ms, flip angle=8°, TFE factor=119, FOV=288×288×175 mm, 175 slices, no gap | 1x 1x 1 | sagittal | 5,3 | Linux  Neurodebian |
| San Diego AdolAN | 1 | General Electric Healthcare | Signa Excite | 3T | SPGR, TI=600 ms, TE=min full, flip angle=8°, matrix=256x192 mm, slice thickness=1.2 mm, 170 slices | 1.2x 1.2x 1.2 | sagittal | 5,3 | CentOS  Linux release  7.4.1708 (Core) |
| San Diego sMRI | 1 | General Electric Healthcare | MR750 | 3T | R-FSPGR, TI=600ms, TE=min full, flip angle=8°, slice thickness=1.2 mm, matrix=256x192 mm | 1.2x 1.2x 1.2 | sagittal | 5,3 | Centos  release 5.11 |
| Toronto | 1 | General Electric Healthcare | Echospeed | 1.5T | 3D FSPGR Sequence, TR=8.2 ms, TE=3 ms, TI=650 ms, flip angle=8°, FOV=240 mm, NEX=1, no gap | 0.9x 0.9x 0.9 | sagittal | 6,0 | Linux |
| Utrecht WOFAN | 1 | Philips | Achieva | 3T | 3D TFE Sequence, TR=8.4 ms, TE=3.8 ms, flip angle=8°, TFE factor=119, FOV=288×288×175 mm, 175 slices, no gap | 1x 1x 1 | sagittal | 5,3 | Linux  Neurodebian |

**Table D.** **Optimal hyperparameters found with grid search.** For every classification task, anorexia nervosa (AN) vs. healthy controls (HC) and anorexia nervosa of restricting subtype (AN-R) vs. anorexia nervosa of binge-eating/purging subtype (AN-BP), a linear support vector machine (SVM) model pipeline was built that included confounds subtraction and principal component analysis (PCA) for feature reduction. Two parameters had to be optimized, using grid search and cross-validation: The percent of variance explained by the retained principal components (PCA %Variance), and the SVM regularization parameter (SVM $C$). The searched grid values were: PCA %Variance=[0.5,.55,0.6,0.65,0.7,0.8,0.9], and SVM $C$=[0.05,0.1,0.5,1.0,1.5,2.0]. ComBat-GAM=ComBat with generalized additive models, LSsO=leave-sites-out cross-validation.

| **Classification task** | **PCA %Variance** | **SVM** $\boldsymbol{C}$ |
| --- | --- | --- |
| **AN vs. HC (ComBat-GAM)** | 0.5 | 0.5 |
| **AN vs. HC (LSsO)** | 0.7 | 2.0 |
| **AN-BP vs. AN-R (ComBat-GAM)** | 0.9 | 0.5 |
| **AN-BP vs. AN-R (LSsO)** | 0.9 | 2.0 |

**Table E.** **Comparison of proportions of infranormal and supranormal z-scores between the groups anorexia nervosa of restricting subtype (AN-R) vs. anorexia nervosa of binge-eating/purging subtype** **(AN-BP).** Comparison of proportions of infranormal z-scores (z<−1.96) and supranormal z-scores (z>1.96) based on the centile brain public models [2, 18, 19] between AN-BP and AN-R. Group differences in the proportion of individuals with supra- or infranormal z-scores were examined using the 2-proportions z-test. After false discovery rate (FDR) correction for multiple comparisons at *q*<.05, the number of supra- or infranormal z-scores did not differ between groups in any region. Reported are regions for which a difference was found at a lenient threshold of uncorrected p<.05. “+”=supranormal z-scores, “−”=infranormal z-scores, Z-Test=two-proportion z-test, L=left hemisphere, R=right hemisphere, thickavg=average cortical thickness, surfavg=average cortical surface area, bankssts=banks of the superior temporal sulcus.

| Index | AN-R + | AN-R - | AN-BP + | AN-BP - | Z-Test  + | Z-Test - |
| --- | --- | --- | --- | --- | --- | --- |
| R thalamus | 1.92 | 9.11 | 1.48 | 3.70 | 0.7407 | 0.0415 |
| R putamen | 0.48 | 15.35 | 0.74 | 7.41 | 0.7198 | 0.0186 |
| L pallidum | 1.68 | 11.99 | 3.70 | 5.93 | 0.1608 | 0.0458 |
| L nucleus accumbens | 0.72 | 14.63 | 3.70 | 14.07 | 0.0117 | 0.8737 |
| L precentral thickavg | 1.20 | 4.56 | 0.00 | 9.63 | 0.2012 | 0.0284 |
| L precuneus thickavg | 0.24 | 14.39 | 0.74 | 7.41 | 0.3998 | 0.0341 |
| R inferiorparietal thickavg | 0.72 | 6.23 | 2.96 | 8.89 | 0.0429 | 0.2898 |
| R insula thickavg | 6.95 | 0.48 | 1.48 | 0.74 | 0.0164 | 0.7198 |
| L bankssts surfavg | 1.92 | 2.64 | 5.19 | 2.22 | 0.0425 | 0.7895 |
| L parsopercularis surfavg | 3.84 | 0.96 | 4.44 | 3.70 | 0.7538 | 0.0286 |
| L parstriangularis surfavg | 3.36 | 1.68 | 3.70 | 5.19 | 0.8478 | 0.0243 |
| L posteriorcingulate surfavg | 3.60 | 0.96 | 4.44 | 3.70 | 0.6547 | 0.0286 |
| R caudalmiddlefrontal surfavg | 4.80 | 2.64 | 0.74 | 2.96 | 0.0323 | 0.8400 |
| R inferiorparietal surfavg | 2.88 | 1.92 | 0.00 | 5.19 | 0.0463 | 0.0425 |
| R pericalcarine surfavg | 1.20 | 1.92 | 2.22 | 5.19 | 0.3873 | 0.0425 |

**Table F.** **Percentages of participants with a significantly high number of supra/-infranormal z-scores in any of a cortical thickness (CT), cortical surface area (SA), and subcortical volume (SV) metrics in each group, and compared between anorexia nervosa (AN) vs. healthy controls (HC) and anorexia nervosa of restricting subtype (AN-R) vs. anorexia nervosa of binge-eating/purging subtype** **(AN-BP).** “+”=supranormal z-scores, “−”=infranormal z-scores, χ=chi-square statistic, p=p-value.

|  | AN | HC | AN-BP | AN-R | AN vs. HC | | AN-BP vs. AN-R | |
| --- | --- | --- | --- | --- | --- | --- | --- | --- |
|  |  |  |  |  | χ | p | χ | p |
| CT+ | 26.49% | 20.70% | 21.48% | 28.06% | 5.72 | .017 | 1.94 | .163 |
| CT- | 14.56% | 12.58% | 10.37% | 15.59% | 0.92 | .338 | 1.86 | .173 |
| SA+ | 14.04% | 16.51% | 11.85% | 14.63% | 1.33 | .250 | 1.22 | .268 |
| SA- | 4.91% | 4.60% | 5.93% | 4.80% | 0.02 | .895 | 0.18 | .672 |
| SV+ | 5.61% | 10.55% | 5.93% | 5.28% | 9.57 | .002 | 0.44 | .502 |
| SV- | 30.00% | 13.13% | 25.19% | 30.94% | 55.24 | <.001 | 0.09 | .769 |

**Table G.** **Deviance explained by machine learning-based risk scores and confounds for the anorexia nervosa (AN) vs. healthy controls (HC) classification.** For each classification pipeline we report the proportion of deviance explained exclusively by model predictions ($\Delta D_{p}^{2}$), exclusively by confounds ($\Delta D_{c}^{2}$), or by both model predictions and confounds ($\Delta D_{p\cap c}^{2}$) using the a posteriori method proposed by Dinga et al. [28]. Explained deviance of model predictions beyond confounds was significant for both the ComBat with generalized additive models (ComBat-GAM) and leave-site-out cross-validation (LSsO) pipelines. We considered both age and intracranial volume (ICV) as confounds. Significance of $\Delta D_{p}^{2}$ was assessed using permutation tests. *p<0.05, **p<0.01, ***p<0.001, n.s. not significant.

|  | | $\Delta D_{p}^{2}$ | $\Delta D_{c}^{2}$ | $\Delta D_{p\cap c}^{2}$ |
| --- | --- | --- | --- | --- |
|  |  | |  |  |
| ComBat-GAM | | 0.245*** | 0.008 | 0.003 |
| LSsO | | 0.195*** | 0.052 | -0.034 |

**Figures**

**
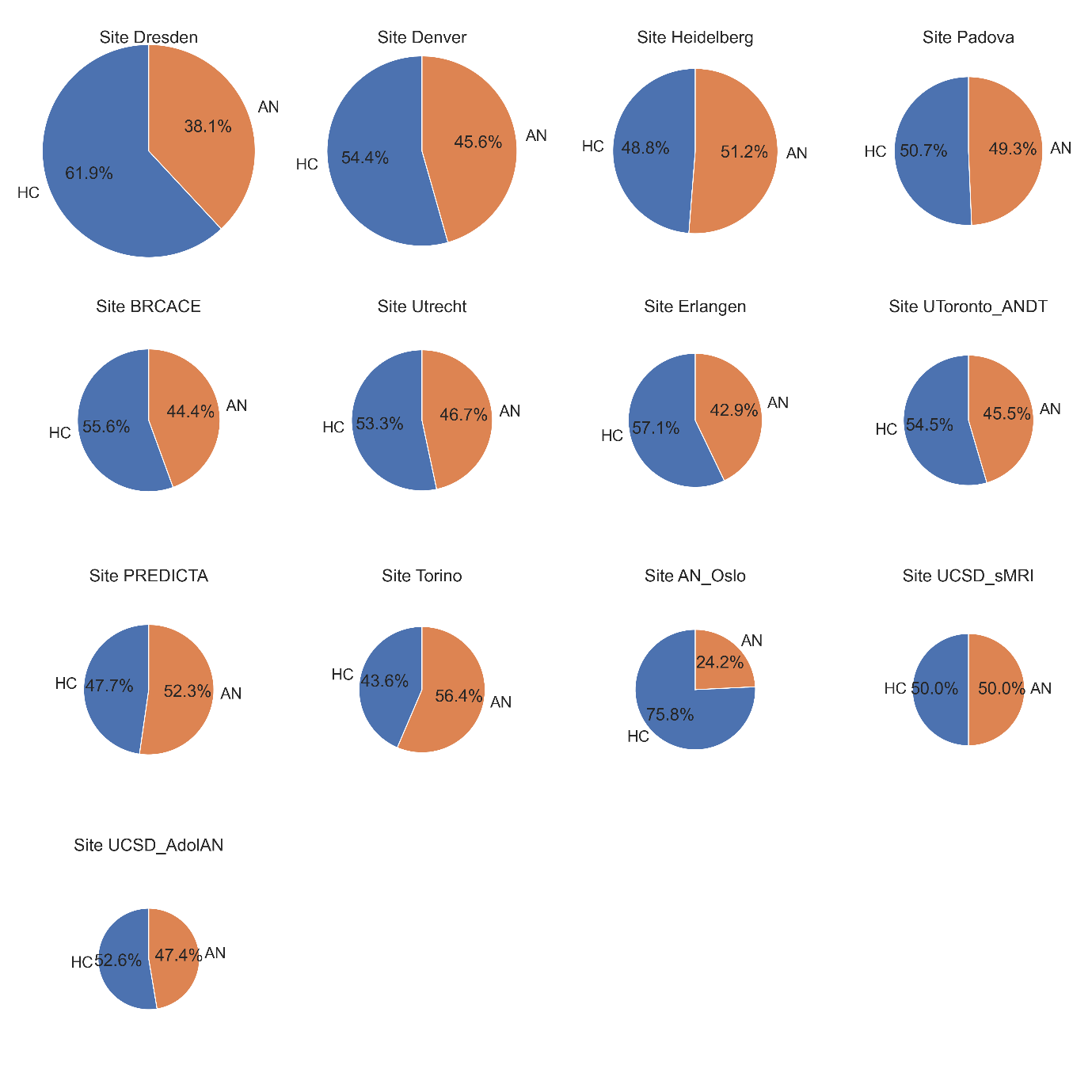
**

**Fig A. Proportion of participants with anorexia nervosa (AN) and healthy controls (HC) per site.** The radius of each circle is proportional to the log_e_ of the total number of participants in the corresponding site.


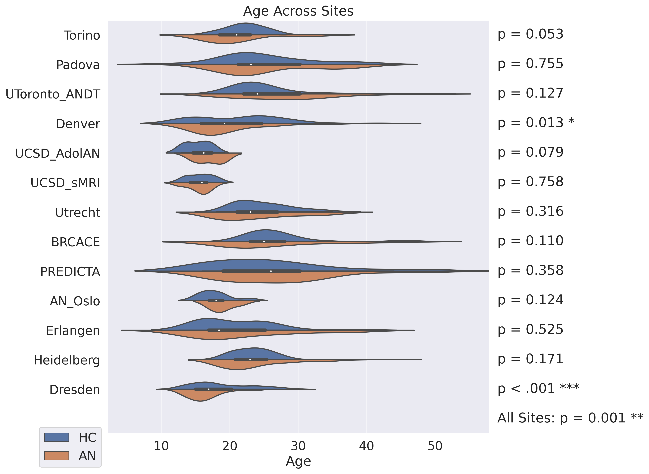

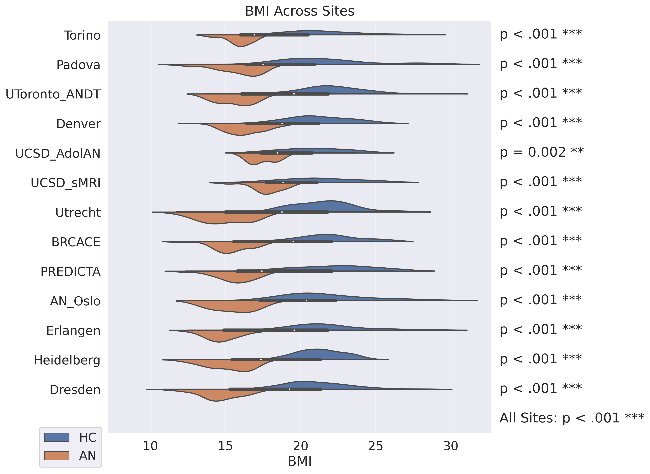


**Fig B. Distribution of age (left) and BMI (right) for anorexia nervosa (AN) vs. healthy controls (HC) analyses in each site.** Given the non-normality of the age and body mass index (BMI) distributions, Mann-Whitney tests were used to assess the significance of the group differences within each site. p=p-value. *p<0.05, **p<0.01, ***p<0.001.


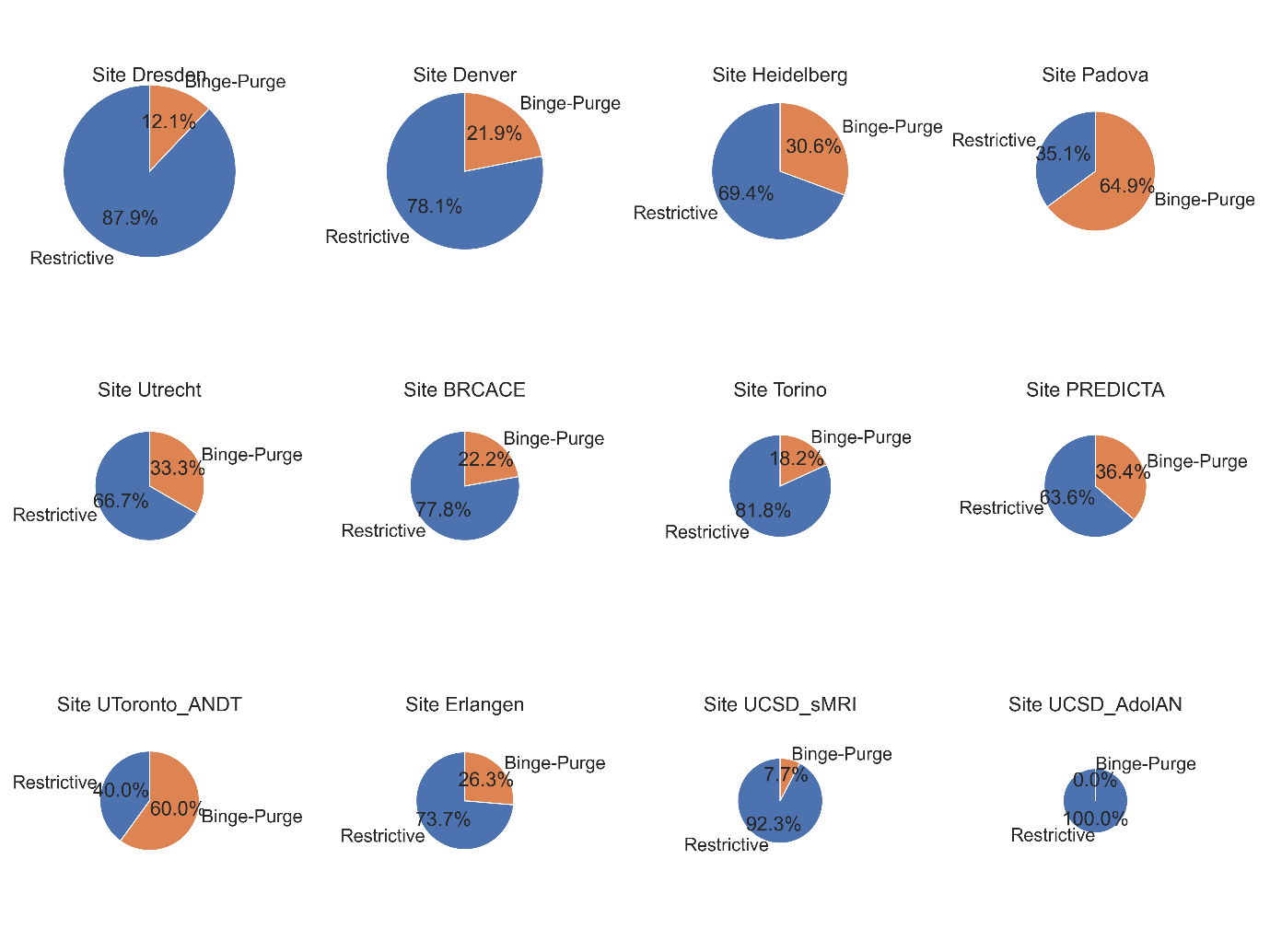


**Fig C. Proportion of participants with anorexia nervosa of the restricting subtype (AN-R) and anorexia nervosa of the binge-eating/purging** **subtype (AN-BP) in the anorexia nervosa (AN) group per site.** The radius of each circle is proportional to the log_e_ of the total number of participants with AN in the corresponding site.

**
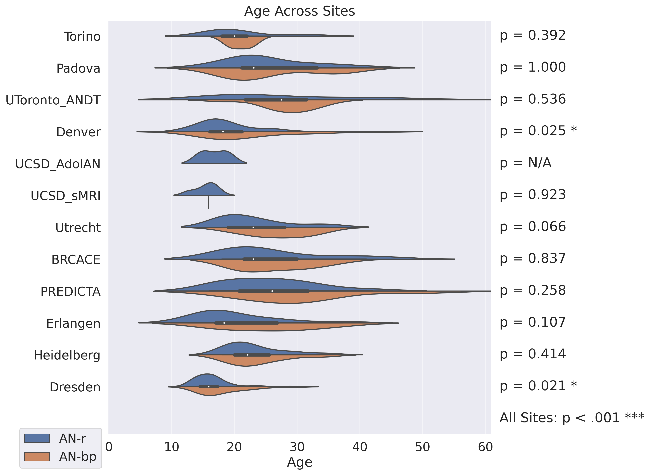

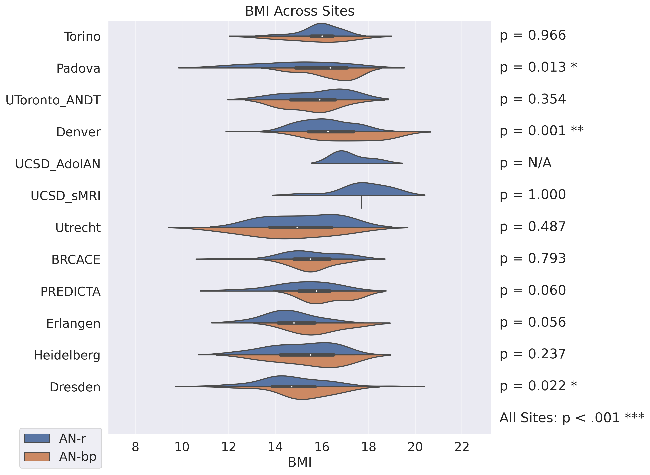
**

**Fig D. Distribution of age (left) and body mass index (BMI, right) anorexia nervosa of the restricting subtype (AN-R) and anorexia nervosa of the binge-eating/purging** **subtype (AN-BP) analyses in each site.** Given the non-normality of the age and BMI distributions, Mann-Whitney tests were used to assess the significance of the group differences within each site. p=p-value. *p<0.05, **p<0.01, ***p<0.001.


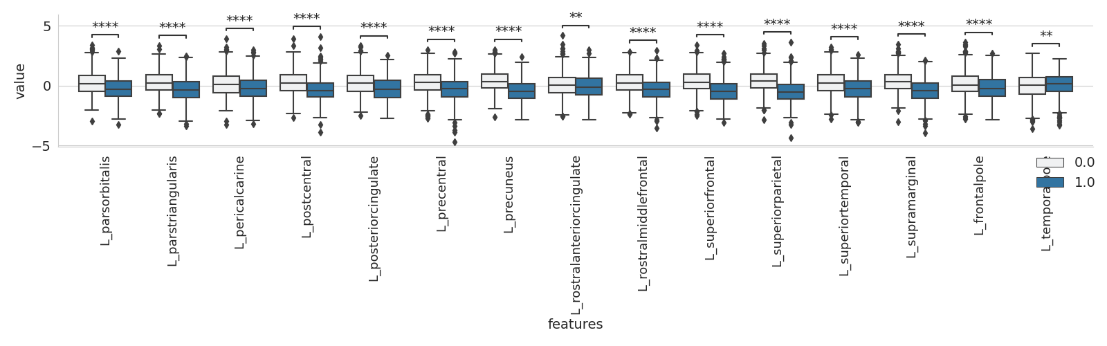

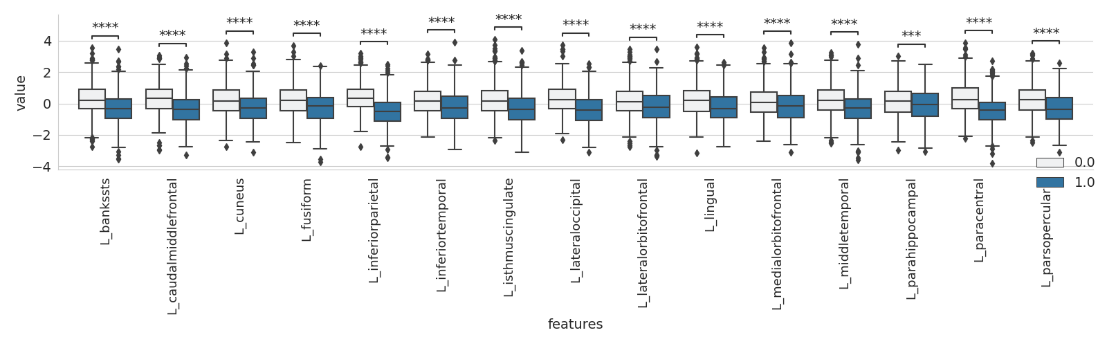


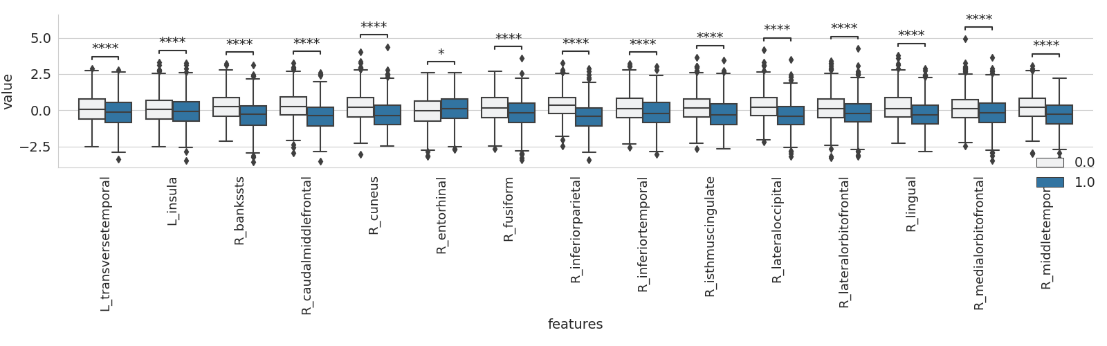


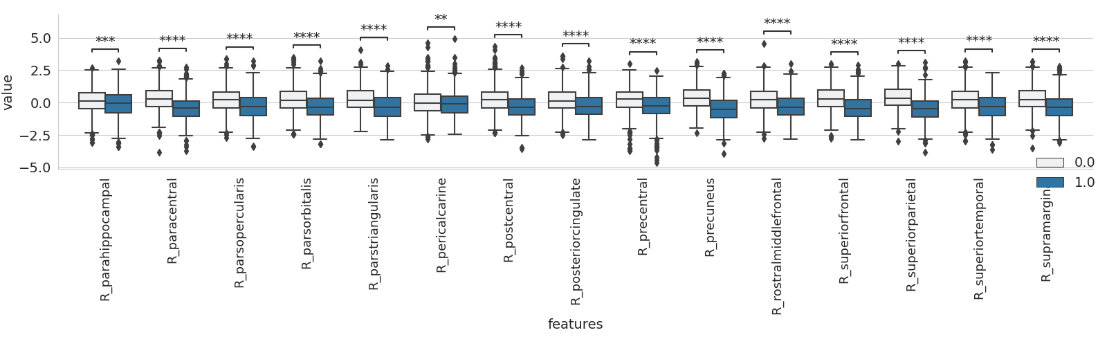


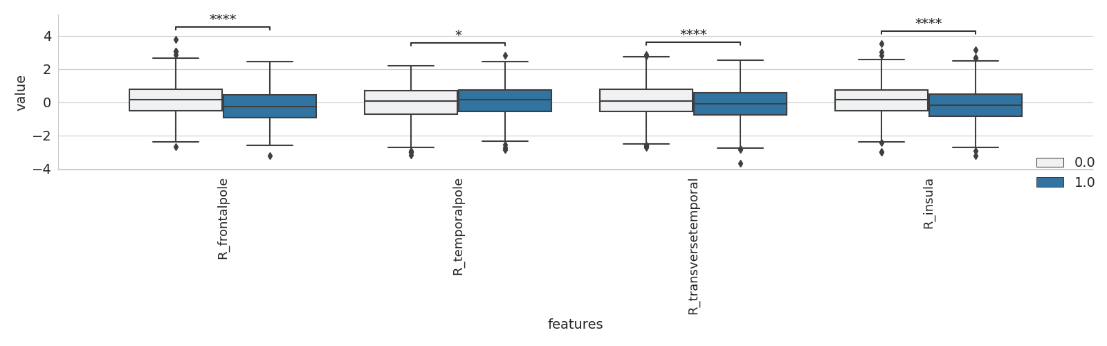


**Fig E.** **Univariate anorexia nervosa (AN, 1) vs. healthy controls (HC, 0) comparisons of cortical thickness (CT).** Box plots show mean-centered and standard deviation-scaled values for each measure. The center line indicates the median, the box limits the interquartile range (25^th^–75^th^ percentile), and the whiskers the most extreme data points within 1.5 times the interquartile range; points beyond the whiskers are shown as outliers. Sites effects were harmonized using ComBat with generalized additive models (ComBat-GAM), then comparisons were estimated within a general linear model with age (including quadratic terms) as a covariate. Plots show results significant after a false discovery rate (FDR) correction at *q*<.05. *q<.05, **q<.01, ***q<.001, ****q<.0001, L=left hemisphere, R=right hemisphere.

**
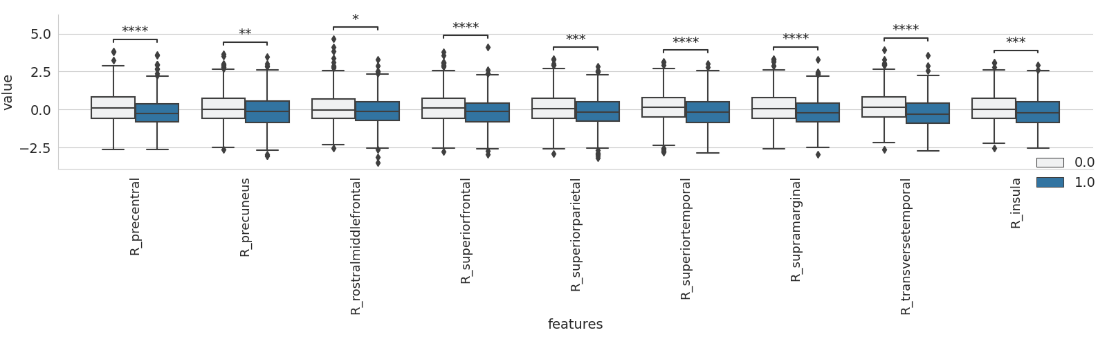

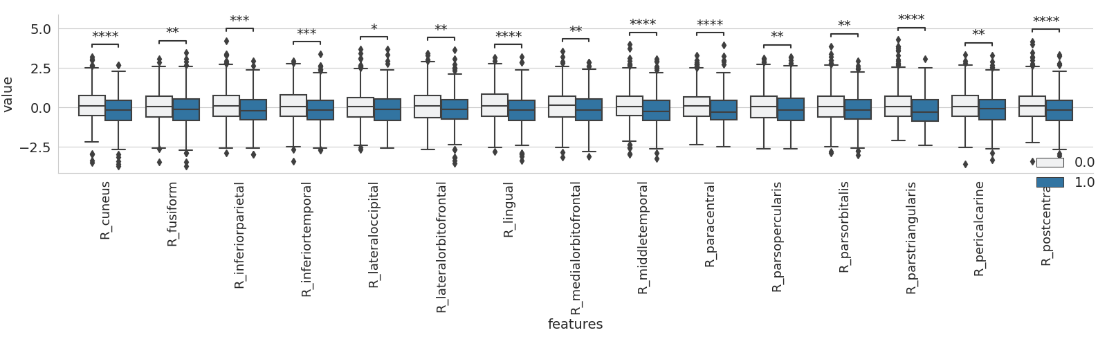

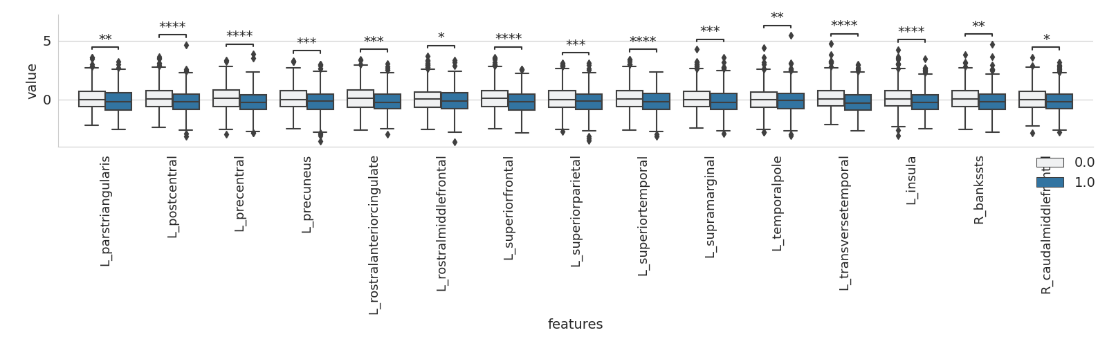

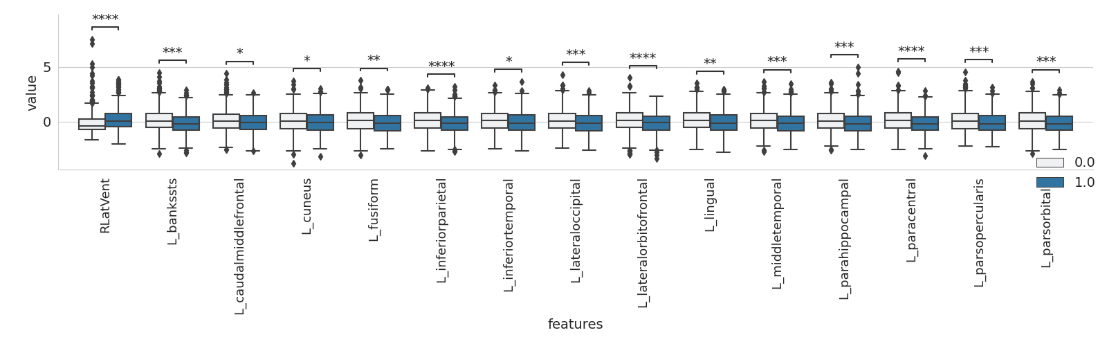

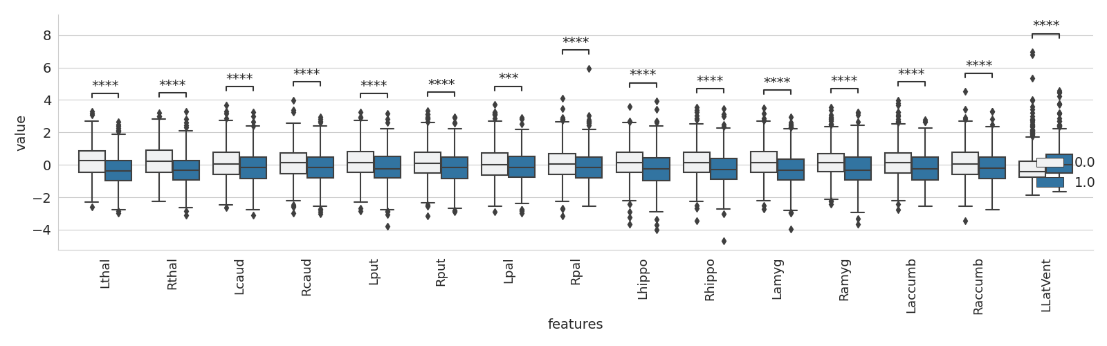

Fig F.** **Univariate anorexia nervosa (AN, 1) vs. healthy controls (HC, 0) comparisons for cortical surface area (SA) and volumes of subcortical gray matter (GM) nuclei.** Box plots show mean-centered and standard deviation-scaled values for each measure. The center line indicates the median, the box limits the interquartile range (25^th^–75^th^ percentile), and the whiskers the most extreme data points within 1.5 times the interquartile range; points beyond the whiskers are shown as outliers. Sites effects were harmonized using ComBat with generalized additive models (ComBat-GAM), then comparisons were estimated within a general linear model with age (including quadratic terms) and intracranial volume as covariates. Plots show results significant after a false discovery rate (FDR) correction at *q:* *<.05, **<.01, ***<.001, ****<.0001, thal=thalamus, caud=caudate nucleus, put=putamen, pal=pallidum, hippo=hippocampus, amyg=amygdala, accumb=nucleus accumbens, LatVent=lateral ventricle, L=left hemisphere, R=right hemisphere.


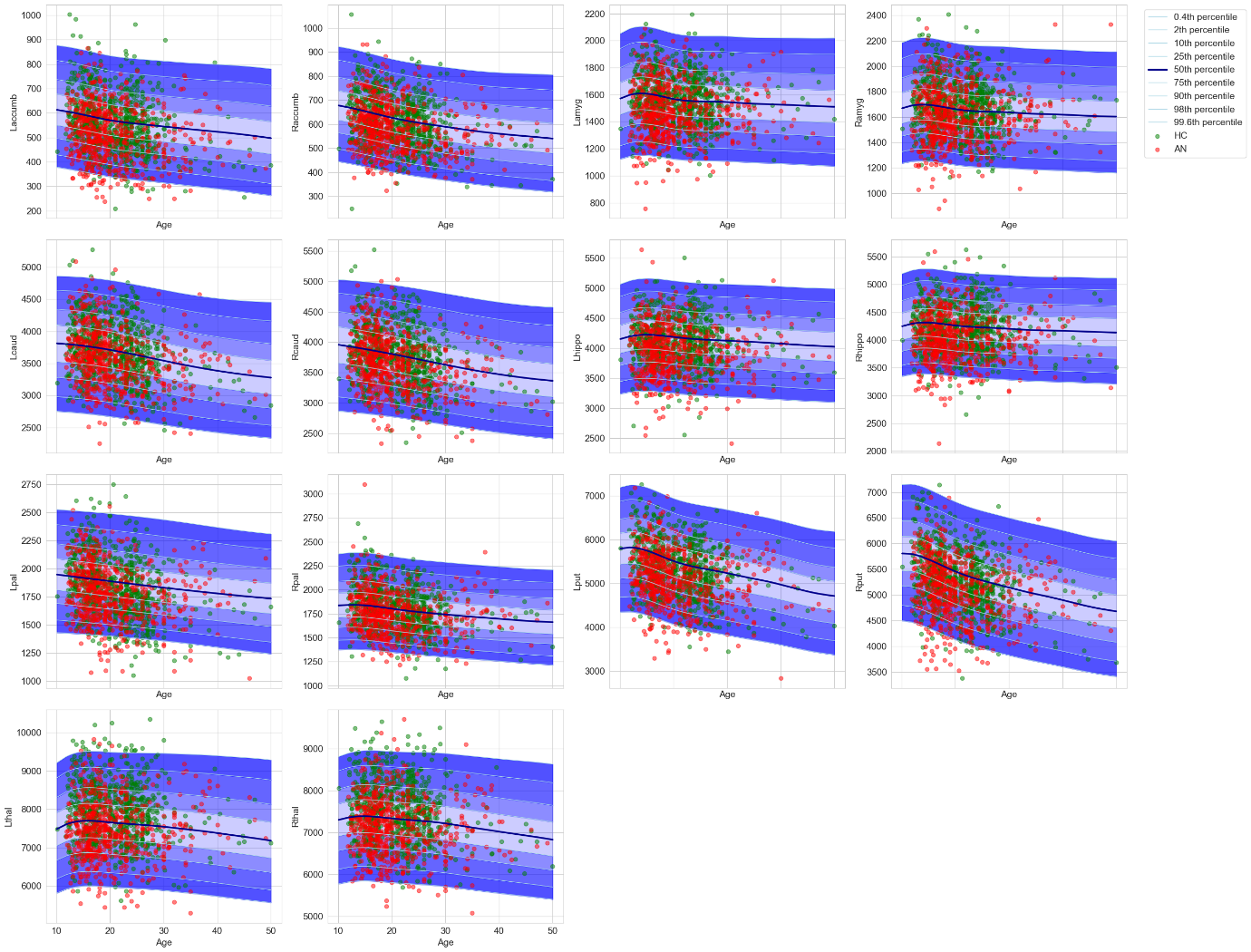


**Fig G. Individual variation for volumes of subcortical nuclei in participants of the healthy control (HC, green) and anorexia nervosa (AN, red) groups superposed to percentile curves computed based on the centile brain public normative models** [2, 18, 19]. These models estimate expected values and normative ranges for brain phenotypes, based on age, sex, mean cortical thickness, total gray matter volume, and cortical surface area. thal=thalamus, caud=caudate nucleus, put=putamen, pal=pallidum, hippo=hippocampus, amyg=amygdala, accumb=nucleus accumbens, L=left hemisphere, R=right hemisphere.


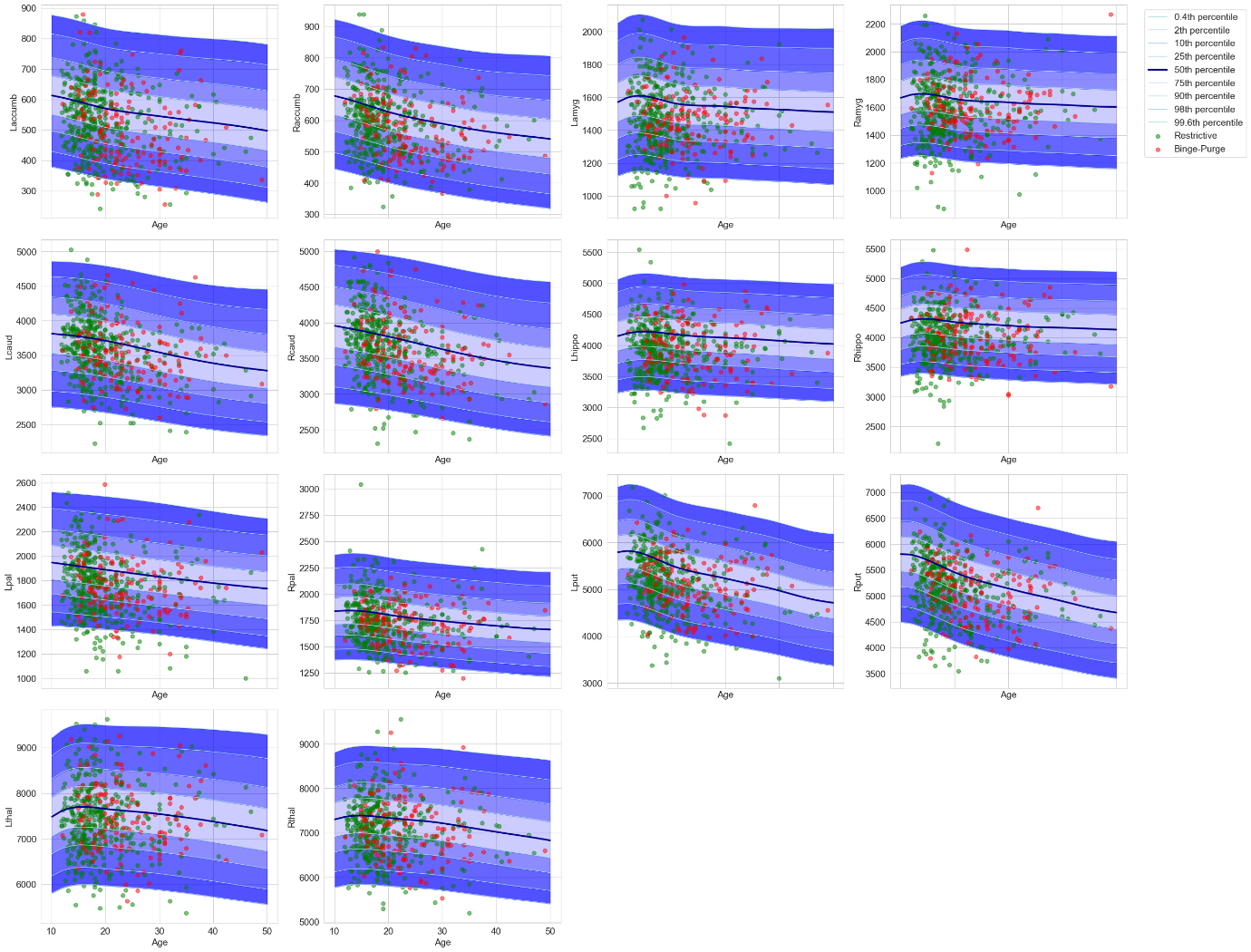


**Fig H.** **Individual variation for volumes of subcortical nuclei in participants of the anorexia nervosa of restricting subtype (AN-R, green) and anorexia nervosa of binge-eating/purging subtype (AN-BP, red) groups superposed to percentile curves computed based on the centile brain public normative models** [2, 18, 19]. These models estimate expected values and normative ranges for brain phenotypes, based on age, sex, mean cortical thickness, total gray matter volume, and cortical surface area. thal=thalamus, caud=caudate nucleus, put=putamen, pal=pallidum, hippo=hippocampus, amyg=amygdala, accumb=nucleus accumbens, L=left hemisphere, R=right hemisphere.


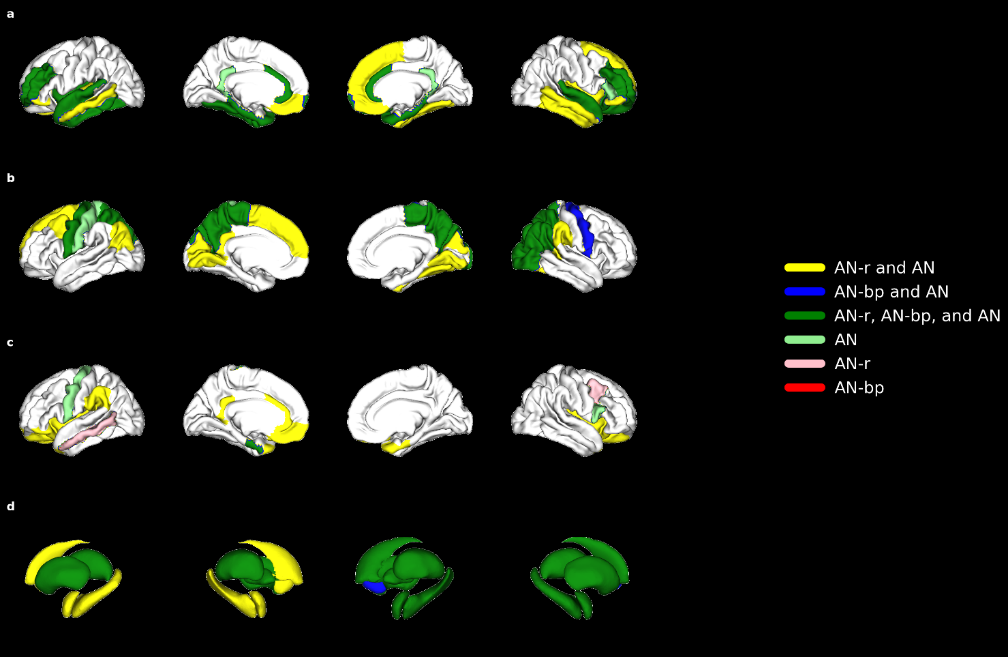
**Fig I.** **Overlap of regions with a percentage of z-scores based on the normative model from the CentileBrain Project, an initiative of ENIGMA's (Enhancing NeuroImaging Genetics through Meta-Analysis) Lifespan Working Group, significantly higher than expected across anorexia nervosa (AN) subtypes.** Colored regions indicate areas where the percentage of supra-/infranormal z-scores exceeded the expected threshold based on the binomial distribution and corrected across measures using an FDR correction *q*<.05, in the whole AN group or in subgroups. For none of the metrics shown the difference in percentage between anorexia nervosa of restricting subtype (AN-R) and anorexia nervosa of binge-eating/purging subtype (AN-BP) was significant. Shown are (a) percentages of supranormal z-scores for cortical thickness (CT) metrics, (b) percentages of infranormal z-scores for CT metrics, (c) percentages of supranormal z-scores for cortical surface area (SA) metrics, and (d) percentages of infranormal z-scores for subcortical volumes (SV) metrics. For no regional metrics of SA the percentage of infranormal z-scores and for no regional metrics of SV the percentage of supranormal z-scores were significantly higher than expected in the normative reference after a false discovery rate correction for multiple comparisons. Thresholds for supra-/infranormal percentages for CT metrics: 4.04%/4.21% (AN), 4.32%/4.56% (AN-R), 6.67%/6.67% (AN-BP). Thresholds for supranormal percentages for SA measures: 4.21% (AN), 4.56% (AN-R), 9.63% (AN-BP). Thresholds for infranormal percentages for SV measures: 4.39% (AN), 4.56% (AN-R), 5.93% (AN-BP).

**
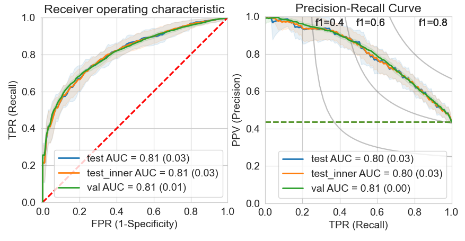
**

**
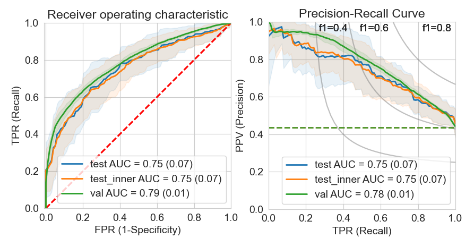

Fig J.** **Test performances achieved by the support vector machines for the anorexia nervosa (AN) vs. healthy controls (HC) classification estimated using nested cross-validation.** Using ComBat with generalized additive models (ComBat-GAM) to harmonize site effects combined with stratified ten-fold cross-validation provides an estimate of the performance on unseen data from the available sites (top) while using Leave Sites Out cross-validation provides an estimate of the performance on unseen data from unseen sites (bottom). The Precision-Recall (a) and corresponding receiver operating characteristic (ROC) (b) curves show test performance averages and standard deviation ranges. The dashed lines represent chance performance. Precision-Recall area under the curve (AUC) was optimized during training. Since Precision is sensitive to group sizes, Precision-Recall curves are not comparable across classification tasks with different proportions of groups. Therefore, also the corresponding ROCs are shown. Permutation tests of the corresponding AUCs showed clear above-chance classification (p<.001). TPR = true positive rate (recall); FPR = false positive rate (1 − specificity); PPV = positive predictive value (precision).

**
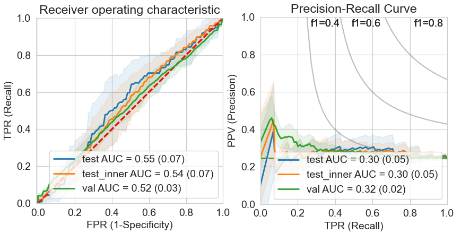
**

**
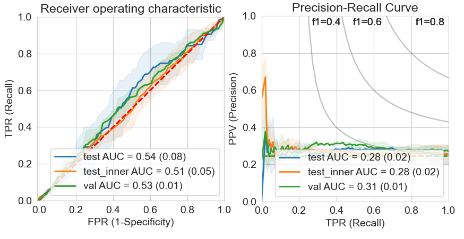
**

**Fig K.** **Test performances achieved by the support vector machines for the anorexia nervosa of the restricting subtype (AN-R) and anorexia nervosa of the binge-eating/purging** **subtype (AN-BP) classification estimated using nested cross-validation.** Using ComBat with generalized additive models (ComBat-GAM) to harmonize site effects combined with stratified ten-fold cross-validation provides an estimate of the performance on unseen data from the available sites (top), while using Leave Sites Out cross-validation provides an estimate of the performance on unseen data from unseen sites (bottom). The Precision-Recall (a) and corresponding receiver operating characteristic (ROC) (b) curves show test performance averages and standard deviation ranges. The dashed lines represent chance performance. Precision-Recall area under the curve (AUC) was optimized during training. Since Precision is sensitive to group sizes, Precision-Recall curves are not comparable across classification tasks with different proportions of groups. Therefore, also the corresponding ROCs are shown. Permutation tests of the corresponding AUCs showed these performances were not superior to chance (p>.12). TPR = true positive rate (sensitivity); FPR = false positive rate (1 − specificity); PPV = positive predictive value (precision).


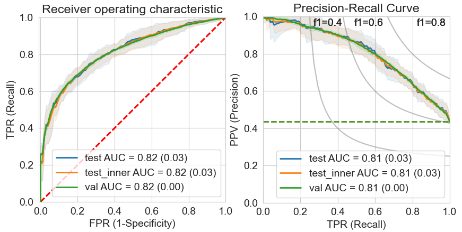


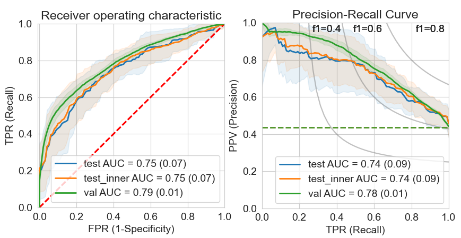


**Fig L.** **Test performances achieved by neural networks for the anorexia nervosa (AN) vs. healthy controls (HC) classification estimated using nested cross-validation.** Using ComBat with generalized additive models (ComBat-GAM) to harmonize site effects combined with stratified ten-fold cross-validation provides an estimate of the performance on unseen data from the available sites (top), while using Leave Sites Out cross-validation provides an estimate of the performance on unseen data from unseen sites (bottom). The Precision-Recall (a) and corresponding receiver operating characteristic (ROC) (b) curves show test performance averages and standard deviation ranges. The dashed lines represent chance performance. Precision-Recall area under the curve (AUC) was optimized during training. Since Precision is sensitive to group sizes, Precision-Recall curves are not comparable across classification tasks with different proportions of groups. Therefore, also the corresponding ROCs are shown. Permutation tests of the corresponding AUCs showed clear above-chance classification (p<.001). TPR = true positive rate (sensitivity); FPR = false positive rate (1 − specificity); PPV = positive predictive value (precision).


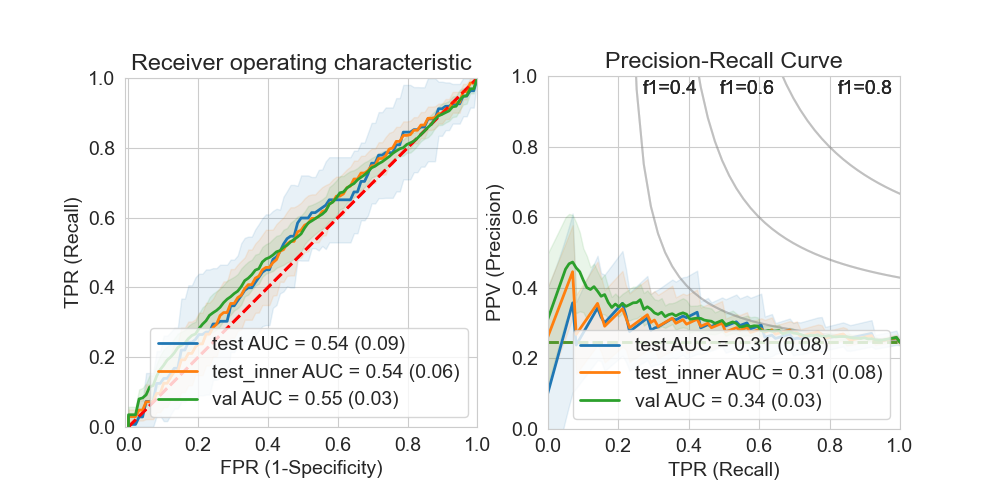


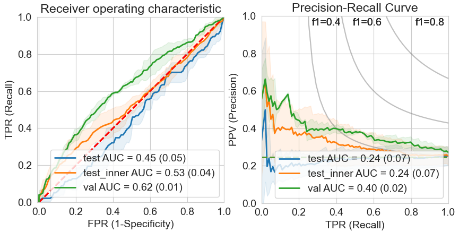


**Fig M.** **Test performances achieved by neural networks for the anorexia nervosa of the restricting subtype (AN-R) and anorexia nervosa of the binge-eating/purging** **subtype (AN-BP) classification estimated using nested cross-validation.** Using ComBat with generalized additive models (ComBat-GAM) to harmonize site effects combined with stratified ten-fold cross-validation provides an estimate of the performance on unseen data from the available sites (top), while using Leave Sites Out cross-validation provides an estimate of the performance on unseen data from unseen sites (bottom). The Precision-Recall (a) and corresponding receiver operating characteristic (ROC) (b) curves show test performance averages and standard deviation ranges. The dashed lines represent chance performance. Precision-Recall area under the curve (AUC) was optimized during training. Since Precision is sensitive to group sizes, Precision-Recall curves are not comparable across classification tasks with different proportions of groups. Therefore, also the corresponding ROCs are shown. Permutation tests of the corresponding AUCs showed these performances were not superior to chance (p>.13). TPR = true positive rate (sensitivity); FPR = false positive rate (1 − specificity); PPV = positive predictive value (precision).
