# Supplementary material for: Brain morphology in Anorexia Nervosa and its subtypes: A multi-cohort study of individual participant data
Source: PLoS Med. 2026 May 20;23(5):e1004809. doi: 10.1371/journal.pmed.1004809 (PMC13215615; doi:10.1371/journal.pmed.1004809)
Supplement: S1 — STROBE checklist for case-control studies. Checklist reproduced from the STROBE Statement (https://www.strobe-statement.org/; von Elm and colleagues, PLoS Med. 2007;4(10):e296. https://doi.org/10.1371/journal.pmed.0040296) under the Creative Commons Attribution 4.0 International License (CC BY 4.0). (DOC) [file pmed.1004809.s002.doc]

**STROBE Statement—Checklist of items that should be included in reports of *case-control studies***

Checklist reproduced from the STROBE Statement (https://www.strobe-statement.org/; von Elm et al., PLoS Med. 2007;4(10):e296. doi:10.1371/journal.pmed.0040296) under the Creative Commons Attribution 4.0 International License (CC BY 4.0).

|  | Item No | Recommendation | Page No |
| --- | --- | --- | --- |
| **Title and abstract** | 1 | (*a*) Indicate the study’s design with a commonly used term in the title or the abstract | Title and paragraph ‘Methods and Findings’ in the Abstract |
|  |  | (*b*) Provide in the abstract an informative and balanced summary of what was done and what was found | Paragraph ‘Methods and Findings’ in the Abstract |
| Introduction | | | |
| Background/rationale | 2 | Explain the scientific background and rationale for the investigation being reported | Paragraphs 1-2 in the Introduction |
| Objectives | 3 | State specific objectives, including any prespecified hypotheses | Pages 6-7; Paragraphs 3-5 in the Introduction |
| Methods | | | |
| Study design | 4 | Present key elements of study design early in the paper | Paragraphs 3-5 in the Introduction,  Sections ‘Study samples’, ‘Group Comparisons’, ‘Normative Modeling’, and ‘Machine Learning Classification’ in the Methods |
| Setting | 5 | Describe the setting, locations, and relevant dates, including periods of recruitment, exposure, follow-up, and data collection | Section ‘Study Samples’ in the Methods, Sections A.1-A.3 in S1 Appendix |
| Participants | 6 | (*a*) Give the eligibility criteria, and the sources and methods of case ascertainment and control selection. Give the rationale for the choice of cases and controls | Section ‘Study Samples’ in the Methods, Sections A.1-A.3 in S1 Appendix |
|  |  | (*b*) For matched studies, give matching criteria and the number of controls per case |  |
| Variables | 7 | Clearly define all outcomes, exposures, predictors, potential confounders, and effect modifiers. Give diagnostic criteria, if applicable | Sections ‘Image acquisition and Processing’, ‘Group Comparisons’, ‘Normative Modeling’, and ‘Machine Learning Classification’ in the Methods |
| Data sources/ measurement | 8* | For each variable of interest, give sources of data and details of methods of assessment (measurement). Describe comparability of assessment methods if there is more than one group | Section ‘Study Samples’and ‘Image acquisition and Processing’ in the Methods, Sections A.1-A.6 in S1 Appendix |
| Bias | 9 | Describe any efforts to address potential sources of bias | Subsections ‘Case-control sample (AN vs. HC)’ and ‘Sample of cases with subtype information (AN-R vs. AN-BP)’, second paragraph of Section ‘Group Comparisons’, Subsection ‘Hyperparameters optimization and performance estimation’ in the Methods, Sections A.5, A.6, A.9-A.12 in S1 Appendix |
| Study size | 10 | Explain how the study size was arrived at | Section ‘Study Samples’ in the Methods, Sections A.1, A.2 in S1 Appendix |
| Quantitative variables | 11 | Explain how quantitative variables were handled in the analyses. If applicable, describe which groupings were chosen and why | Sections ‘Group Comparisons’, ‘Normative Modeling’, and ‘Machine Learning Classification’ in the Methods, Sections A.5-A.12 in S1 Appendix |
| Statistical methods | 12 | (*a*) Describe all statistical methods, including those used to control for confounding | Sections ‘Group Comparisons’, ‘Normative Modeling’, and ‘Machine Learning Classification’ in the Methods, Sections A.5-A.12 in S1 Appendix |
|  |  | (*b*) Describe any methods used to examine subgroups and interactions | Sections ‘Group Comparisons’, ‘Normative Modeling’, and ‘Machine Learning Classification’ in the Methods, Sections A.5-A.12 in S1 Appendix |
|  |  | (*c*) Explain how missing data were addressed | Subsection ‘Sample of cases with subtype information (AN-R vs. AN-BP)’ in the Methods |
|  |  | (*d*) If applicable, explain how matching of cases and controls was addressed | Subsections ‘Case-control sample (AN vs. HC)’ and ‘Sample of cases with subtype information (AN-R vs. AN-BP)’ in the Methods |
|  |  | (*e*) Describe any sensitivity analyses | Subsections ‘Classification Pipelines’ and ‘Hyperparameters optimization and performance estimation’, Sections A.5, A.7, A.11 in S1 Appendix |
| Results | | | |
| Participants | 13* | (a) Report numbers of individuals at each stage of study—eg numbers potentially eligible, examined for eligibility, confirmed eligible, included in the study, completing follow-up, and analysed | Subsections ‘Case-control sample (AN vs. HC)’ and ‘Sample of cases with subtype information (AN-R vs. AN-BP)’ in the Methods, Section Demographics in the Results, Sections A.1, A.2, Tables A, B in S1 Appendix. |
|  |  | (b) Give reasons for non-participation at each stage | Subsections ‘Case-control sample (AN vs. HC)’ and ‘Sample of cases with subtype information (AN-R vs. AN-BP)’ in the Methods, Sections A.1, A.2 in S1 Appendix. |
|  |  | (c) Consider use of a flow diagram |  |
| Descriptive data | 14* | (a) Give characteristics of study participants (eg demographic, clinical, social) and information on exposures and potential confounders | Section Demographics in the Results, Sections A.1, A.2, Tables A, B in S1 Appendix. |
|  |  | (b) Indicate number of participants with missing data for each variable of interest | Subsections ‘Case-control sample (AN vs. HC)’ and ‘Sample of cases with subtype information (AN-R vs. AN-BP)’ in the Methods, Tables A, B in S1 Appendix. |
| Outcome data | 15* | Report numbers in each exposure category, or summary measures of exposure | Subsections ‘Case-control sample (AN vs. HC)’ and ‘Sample of cases with subtype information (AN-R vs. AN-BP)’ in the Methods, Tables A, B in S1 Appendix. |

| Main results | | 16 | (*a*) Give unadjusted estimates and, if applicable, confounder-adjusted estimates and their precision (eg, 95% confidence interval). Make clear which confounders were adjusted for and why they were included | Sections ‘Univariate comparisons’, ‘z-scores from CentileBrain normative model’, ‘Machine learning classification’ in the Results, Figures 1-3, Section B, Tables E, F, Figures E-M in S1 Appendix. |
| --- | --- | --- | --- | --- |
|  |  |  | (*b*) Report category boundaries when continuous variables were categorized |  |
|  |  |  | (*c*) If relevant, consider translating estimates of relative risk into absolute risk for a meaningful time period |  |
| Other analyses | 17 | Report other analyses done—eg analyses of subgroups and interactions, and sensitivity analyses | | Section ‘Machine learning classification’ in the Results, Table G, Figures J-M in S1 Appendix |
| Discussion | | | | |
| Key results | 18 | Summarise key results with reference to study objectives | | First paragraph in the Discussion |
| Limitations | 19 | Discuss limitations of the study, taking into account sources of potential bias or imprecision. Discuss both direction and magnitude of any potential bias | | Sixth paragraph in the Discussion |
| Interpretation | 20 | Give a cautious overall interpretation of results considering objectives, limitations, multiplicity of analyses, results from similar studies, and other relevant evidence | | Second to fifth paragraphs in the Discussion |
| Generalisability | 21 | Discuss the generalisability (external validity) of the study results | | Third, fourth and sixth paragraphs in the Discussion |
| Other information | | | | |
| Funding | 22 | Give the source of funding and the role of the funders for the present study and, if applicable, for the original study on which the present article is based | | Acknowledgments and Disclosures and Competing Interests sections |

*Give information separately for cases and controls.

**Note:** An Explanation and Elaboration article discusses each checklist item and gives methodological background and published examples of transparent reporting. The STROBE checklist is best used in conjunction with this article (freely available on the Web sites of PLoS Medicine at http://www.plosmedicine.org/, Annals of Internal Medicine at http://www.annals.org/, and Epidemiology at http://www.epidem.com/). Information on the STROBE Initiative is available at http://www.strobe-statement.org.
