# Supplementary material for: Brain morphology in Anorexia Nervosa and its subtypes: A multi-cohort study of individual participant data
Source: PLoS Med. 2026 May 20;23(5):e1004809. doi: 10.1371/journal.pmed.1004809 (PMC13215615; doi:10.1371/journal.pmed.1004809)
Supplement: Attachment — Submitted filename: Review_Comments_PMEDICINE-D-25-03733R1.docx [file pmed.1004809.s004.docx]

**Review Comments**

**Manuscript Title:** Brain morphology in Anorexia Nervosa and its subtypes: Mega-Analysis, Normative Modelling, and Machine Learning Approaches

The following manuscript provides a mega-analysis of neuroimaging data from 11 international sites to investigate structural differences in those with anorexia nervosa (AN) relative to healthy control. This mega-analysis utilised group-wise differences, evaluation of structural in relation to referential z-scores, and machine learning approaches to investigate brain morphology in AN and their subtypes. Findings confirm those seen in extant literature identifying widespread reductions in grey matter in those with AN relative to HCs, and could be accurately classified using machine learning approaches. This study uses multiple statistical techniques to investigate neuroanatomical nuances in those with AN, and is significantly strengthened by the large sample sizes available and subsequent study power. The methodological approach is clearly and transparently outlined, particularly the fine-tuning of their optimisation parameters for machine-learning-based classification. There are very minor issues regarding referencing, but more importantly I have concerns regarding BMI-based cut-off thresholds to distinguish between study groups and what implications this may have on morphological findings.

A significant proportion of neuroimaging research in anorexia nervosa include studies with limited sample sizes, and the extent and array of data provided within this mega-analysis is a considerable strength of this study, and provides enormous benefit towards the understanding of nuanced variations in brain structure in AN. Providing the authors can address methodological concerns, I have no doubt this study will make a significant contribution to the current literature. Suggestions to bolster this work alongside additional considerations are further outlined below.

| **#** | **Review Comment** | | **Manuscript Segment** | **Location (Page)** |
| --- | --- | --- | --- | --- |
| **Introduction** | | | | |
| 1. | | Minor: the following sentence requires a hyphen for ‘group level’, as well as a space between ‘subtypes’ and ‘[7]’: “In contrast to our previous group level meta-analysis, analyzing individual-level data enables examination of variability related to clinical AN subtypes[7].” | Introduction | P.4 |
| 2. | | To assist prospective readers, I’m wondering if the authors may be able to further elaborate on the distinction between the AN-BP subtype and bulimia nervosa. Recent literature reviewing AN subtypes has debated the classification and operationalisation of AN-BP and AN-R subtypes (*Albracht-Schulte KD, Flynn L, Gary A, Perry CM, Robert-McComb JJ. The Physiology of Anorexia Nervosa and Bulimia Nervosa. InThe Active Female: Health Issues throughout the Lifespan 2023 Feb 26 (pp. 95-117). Cham: Springer International Publishing.*), as well as significant diagnostic crossover and transition between subtypes (*Serra R, Di Nicolantonio C, Di Febo R, De Crescenzo F, Vanderlinden J, Vrieze E, Bruffaerts R, Loriedo C, Pasquini M, Tarsitani L. The transition from restrictive anorexia nervosa to binging and purging: a systematic review and meta-analysis. Eating and Weight Disorders-Studies on Anorexia, Bulimia and Obesity. 2022 Apr;27(3):857-65*.).  I think this debate is important for the author team to acknowledge, and has additional implications on study hypotheses associated with structural morphology of the brain. | Introduction | P.4 |
| 3. | | Similar to the comment above, I think it is pertinent to touch upon previous literature aiming to investigate brain morphological or functional distinctions between AN subtypes (e.g., *Yonezawa H, Otagaki Y, Miyake Y, Okamoto Y, Yamawaki S. No differences are seen in the regional cerebral blood flow in the restricting type of anorexia nervosa compared with the binge eating/purging type. Psychiatry and clinical neurosciences. 2008 Feb;62(1):26-33*.), and how the availability and richness of data present in this study may be more suitable to comprehensively investigate these nuances.  I note that the authors have begun to touch upon this at the bottom of page four within their introduction, but I think that discussion of previous literature would further strengthen the study rationale. | Introduction | P.4 |
| 4. | | Minor: There is an issue with plurality in the following text: “(e.g., cortical thickness (CT) in specific brain region)” | Introduction | P.5 |
| **Methods** | | | | |
| 1. | | Minor: As the authors have already abbreviated ‘the ENIGMA Eating Disorder Working Group’ to read as ‘The ENIGMA ED working group’ within the Introduction, I would suggest they remain consistent in use of the abbreviation within the methods and subsequent text. | Methods – Study samples | P.6 |
| 2. | | Could the authors elaborate on the BMI cut-offs used for adults within their inclusion and exclusion criteria? ICD-11 classifications characterise AN as having a BMI falling below 18.5 kg/m^2^, while DSM-5 classifications include BMI ranges, which mark AN severity as mild (BMI ≥ 17 kg/m^2^), moderate (BMI 16–16.99 kg/m^2^), severe (BMI 15–15.99 kg/m^2^) or extreme (BMI < 15 kg/m^2^; *Himmerich H, Treasure J. Anorexia nervosa: diagnostic, therapeutic, and risk biomarkers in clinical practice. Trends in Molecular Medicine. 2024 Apr 1;30(4):350-60*.).  Considering the present range of <17.5 for AN falls between these classifications, it would benefit readers for the authors to rationale this cut-off. This includes elaborating upon including adults with a BMI below a healthy range within their HC group. I hope the authors have a solidified rationale for this decision, considering a significant portion of neuroanatomical research into AN has failed to accurately distinguish brain atrophy associated with malnutrition from AN-specific morphological changes. | Methods – Study samples | P.6 |
| 3. | | I think the reader would benefit from a short, sentence-long description as to how AN subtypes were classified prior to referencing the Supplementary Methods. | Methods – Sample of cases with subtype information (AN-R vs. AN-BP) | P.6 |
| 4. | | Which MRI manufacturers and models were utilised for T1-weighted image acquisition across sites? Retrospectively, I can see that these are provided within Table S3, but can the authors make mention of this within the metnhods? | Methods – Image acquisition and processing | P.7 |
| 5. | | Minor: The reference for Pomponio et al. (2020) is not numbered, and references Bahnsen et al. 2022 and Bernardoni et al. 2016 seem to use a different referencing style than the rest of the manuscript. | Methods – Group comparisons | P.7 |
| 6. | | Minor: Should CentileBrain be appropriately referenced at first mention? | Methods – Normative modeling | P.8 |
| 7. | | Could the authors include mention of using 2-proportion z-tests to assess proportions of infranormal or supranormal z-scores between AN-BP and AN-R? | Methods – Normative modeling | P.8 |
| 8. | | Minor: Arold et al. (2023) should be appropriately numbered to remain consistent with referencing style. | Methods – Machine learning classification | P.8 |
| 9. | | Minor: Under ‘Explainable AI’, “AI” should be spelled out in full at first mention | Methods – Machine learning classification | P.10 |
| **Results** | | | | |
| 1. | | Minor: the AN subtypes should be abbreviated in the following sentence for consistency: “The age difference in subtypes may reflect the more typical transition from the restricting to the binge-eating/purging subtype over time [14, 43, 44].” | Results - Demographics | P.10 |
| 2. | | Minor: Please remain consistent with spelling of “Mean (SD)”; at times it is presented with a space before brackets (e.g., ‘Mean (SD)’), and during other times no space is present (e.g., ‘Mean(SD)’). | Results – Univariate comparisons | P.10 |
| 3. | | To provide readers with a bit of context, could the authors elaborate on what would generally be considered appropriate or high performance for classifier models, particularly in reference to ROC-AUC values? I think the provision of referential values would ease reader interpretation of these findings, particularly those less well-versed in machine learning techniques. | Results – Machine learning classification | P.14 |
| **Discussion** | | | | |
| 1. | | Minor: Is it necessary to re-introduce the abbreviations for cortical thickness, subcortical volume and cortical surface area within this section? | Discussion | P.16 |
| 2. | | Minor: Walton et al. (2022) should be appropriately numbered, and the year should be included within the following portion of text: “the effect sizes were comparable (d=-0.55(0.21) vs. d=-0.57(0.20) in Walton et al.), while […]” | Discussion | P.16 & P.17 |
| 3. | | Is there extant literature that the authors are able to draw on regarding their interpretation of findings stemming from their analysis of z-scores from the CentileBrain group? Have other studies utilised this approach, and can the authors explicitly acknowledge this? The authors are making multiple interpretations in this section that, without reference to adequate evidence, borders on speculation rather than interpretation. | Discussion | P.17 |
| 4. | | The following text should fall within the methods or Supplementary Methods section, rather than within the discussion: “To address site-related variability, we implemented two complementary strategies. In the first, we applied ComBat-GAM to explicitly remove site effects prior to model training [33]. In the other, site correction was not performed; instead, we used a leave-site-out cross-validation (LSsO) strategy, ensuring that all test data came from sites unseen during training.” | Discussion | P.17 |
| **Figures/Tables** | | | | |
| 1. | | The following text should be included within the methods or Supplementary Methods, rather than within the figure legend: “To assess the importance of each feature for the classification, we computed the Pearson correlation coefficient between each feature and the machine learning-based risk score (Haufe et al., 2014)”. As a minor point, the reference should also be appropriately numbered as per the recommended style. | Figure 3. | P.15 |
| **Supplementary Methods** | | | | |
| 1. | | The authors note here that participants were classified as having AN with a BMI <18.5, but this value is noted as 17.5 within the main manuscript. Could the authors clarify? | 1.2 Study Criteria | P.29 |
| 2. | | Table S3-Table S7 are missing appropriate abbreviations sections. | Table S3-Table S7 | P.45-P.48 |
| 3. | | Similar to the supplementary tables, Figure S1-Figure S13 inconsistently display footnotes to clarify included abbreviations. | Figure S1-Figure S13 | P.49-P.61 |
