# Supplementary material for: Brain morphology in Anorexia Nervosa and its subtypes: A multi-cohort study of individual participant data
Source: PLoS Med. 2026 May 20;23(5):e1004809. doi: 10.1371/journal.pmed.1004809 (PMC13215615; doi:10.1371/journal.pmed.1004809)
Supplement: Attachment — Submitted filename: Bernardoni_et_al_Response2Reviewers.docx [file pmed.1004809.s006.docx]

**Comments from the editorial team**

Please revise the abstract to address the following points:

- Intro: please explain more clearly how this study addresses the issue identified in the final sentence of the Background section. This explanation should be incorporated as the new concluding sentence of the Background portion.
- Methods and Findings: The key novel results could be more clearly described in the Methods and Findings section of the abstract. Please revise accordingly.

*We have revised the abstract as requested. In the Background section, we now more clearly state how the present study addresses the limitation noted in the final sentence by explicitly outlining our extension beyond group-level effects. In the Methods and Findings section, we have also revised the text to describe the key novel results more clearly, adding more detailed and quantitative reporting of the main findings.*

**Comments from the reviewers**

**Reviewer #1**: "Brain morphology in Anorexia Nervosa and its subtypes: Mega-Analysis, Normative Modelling, and Machine Learning Approaches" analyzed data from 11 international sites in the ENIGMA Eating Disorders Working Group, in particular cortical thickness and subcortical volumes in anorexia nervosa (AN) subjects versus healthy controls (HC). Three modelling techniques (statistical mega-analysis, normative modelling, machine learning) were applied. In general, they all found significant (and discriminative) differences between the AN and HC groups with respect to various quantitative measures. This may enable the classification of AN subjects with improved accuracy as compared to MRI-based psychiatric classification.

While the presented evidence is largely convincing, some issues might be considered:

*We thank the reviewer for the careful evaluation of our manuscript and for the positive and balanced assessment of our work.*

1. In the Introduction section, it is stated that a previous prospective harmonized meta-analysis from the ENIGMA Eating Disorders (ED) Working Group revealed sizeable and widespread gray matter (GM) reductions associated with low weight and body mass index (BMI) in AN. It might be briefly clarified whether these correlations were likewise observed with naturally lower weight and BMI subjects (that did not have AN), if possible.

*We agree with the reviewer that examining gray matter alterations associated with low BMI in the absence of AN would be highly informative for disentangling effects related to underweight from those specific to the disorder. However, only few studies have addressed related questions, in part due to the difficulty of recruiting individuals with persistently low BMI without AN. Existing evidence suggests that gray matter reductions can also be observed outside the context of AN. In a recent study, underweight healthy controls showed reduced surface area in the right orbitofrontal cortex and left insula, as well as lower volumes of the bilateral amygdala, right hippocampus, and left thalamus; BMI-matched patients with AN exhibited additional reductions, including smaller subcortical volumes (bilateral pallidum and caudate, left putamen, right thalamus) and cortical thinning in default-mode network regions (bilateral inferior parietal lobule, right precuneus, posterior cingulate cortex, left cuneus), consistent with potential AN-specific alterations [1]. Furthermore, gray matter alterations have been reported in early-onset restrictive eating disorders in very young samples (<13 years), with regional patterns differing between early AN and avoidant/restrictive food intake disorder [2], and widespread cortical thickness reductions have been observed in children aged 9–11 years in the Generation R cohort [3]. Although age and BMI-SDS in these samples differ substantially from those in the present study, these findings suggest that at least part of the gray matter reductions reported in our previous ENIGMA meta-analysis may be attributable to low BMI or undernutrition rather than AN per se. We have added these references in the introduction (lines 109-111).*

2. In the Study samples subsection, the female-only exclusion criteria might be briefly substantiated, and the diagnosis of AN (as opposed to HC) briefly described.

*We thank the reviewer for this suggestion. We have now briefly substantiated the female-only exclusion criterion in the Study samples subsection. Specifically,* *AN has a markedly higher prevalence in females (e.g., lifetime prevalence up to ~4% in females vs ~0.3% in males* [4]*), and across ENIGMA-ED sites the number of male participants was too small to support adequately powered, sex-stratified analyses; therefore, restricting the sample to females reduced heterogeneity and avoided confounding by sex-related differences in brain morphology (lines 183-185). We also added a brief description of the AN diagnostic criteria (DSM-5), i.e.,* *restriction of energy intake leading to significantly low body weight, intense fear of gaining weight (or persistent behavior interfering with weight gain), and disturbance in body weight/shape experience or undue influence on self-evaluation (lines 172-177).*

3. In the Case-control sample (AN vs. HC) subsection, the selection of cases and any associated matching of AN to HC cases, might be briefly described.

*We have now clarified in the Study Sample subsection that* *patients with AN and healthy controls were selected within each site according to standardized inclusion and exclusion criteria (lines 167-169), and in the Case-Control Sample subsection that cases and controls were age-matched within site (lines 194-195).*

4. In the Group Comparisons section, it is stated that univariate mega-analyses were performed. It might be clarified as to whether any covariates/confounds (e.g. age, weight) were considered and/or applied to the analyses, or if these were assumed to be addressed by the ComBat-GAM harmonization process (as detailed in Supplementary 1.5).

*We apologize this detail was not expressed clearly. While ComBat-GAM was used to remove site effects in such a way to preserve within each site the dependence related to confounds, the effect of confounds was removed in a subsequent step by including them (age, weight, intracranial volume for surface area and subcortical volume analyses and BMI for subtype analyses) as covariates in the GLM used to test for group differences. We have improved the description of the statistical methods used to make this point clearer (lines 225-231). Importantly, we have made the code used for the analyses available on OSF (project: osf.io/bvryd; view-only link: osf.io/bvryd/?view_only=8e0839869b83400f93418ed24f8237bf).*

5. In the Normative modeling section, the CentileBrain brain morphometry models are stated to be sex-specific, but derived from an ethnoracial diverse sample of >37,000 individuals aged 5-90 years. It might be briefly clarified as to the extent to which this reference population coincides with that for the collated ENIGMA EDWG data, especially given the relatively lower ages of the study population (from Table S1 etc.)

*Although the CentileBrain reference models were derived from a wide age range (5–90 years), normative z-scores are computed conditional on age and sex, such that each participant is compared to an age- and sex-matched reference. The age range of the ENIGMA EDWG sample falls well within the supported range of the normative models. Moreover, in the foundational paper for the CentileBrain model* [5] *it was shown that the accuracy of the CentileBrain models is robust across different age bins (https://pmc.ncbi.nlm.nih.gov/articles/PMC10929064/figure/F6/). The figure is appended below for ease of access:*

*The CentileBrain morphometry reference models were trained on >37,000 healthy individuals aggregated from 87 cohorts spanning Europe, Australia, the USA, South Africa, and East Asia. Because ethnoracial categories are not uniformly defined or reported across contributing cohorts, the overall ethnoracial composition of the reference sample is not consistently documented; nevertheless, given that several major contributing resources (e.g., UK Biobank) are predominantly of European ancestry, the training sample is likely skewed toward European ancestry despite inclusion of non-European cohorts. Accordingly, we cannot quantify the degree of overlap in ethnoracial distribution between the CentileBrain reference population and the collated ENIGMA EDWG data; however, both are plausibly dominated by European-ancestry participants. We have therefore added a statement to the Limitations (lines 556-559) noting the limited availability of harmonized ethnoracial data and the potential implications for generalizability.*

6. For Normative modeling, results for the HC group would be relevant too, and might be presented if possible.

*We agree with the reviewer that conventional case–control comparisons are informative in many settings. However, they address a different question from the one posed in this study and, in the context of normative modelling analyses, they would be methodologically misaligned with our primary objective. Our aim was not to estimate average group differences in the frequence of extreme deviations between ENIGMA ED WG patients and controls, but to characterize regional patterns of normative deviation at the individual level. Normative modelling is explicitly designed for this purpose.*

*The healthy control samples collected alongside the clinical cohorts were not designed to function as a population reference group. One of the major criticisms of case-control designs has been that the control group is typically a convenience sample with limited size and restricted demographic and recruitment characteristics (for example, in our case HC with BMI>30, preterm birth <30 weeks, neurological, developmental or other psychiatric disorders were excluded), and it may not represent the broader distribution of neuroanatomical variation. By design, the normative models already provide the appropriate reference, leveraging large-scale population data to estimate expected variation across the age range while reducing dependency on any single local control sample. A conventional case–control arm would create a parallel inferential framework to the normative analysis not directly comparable to the deviation-based outcomes. Given that the manuscript’s conclusions are framed around individualized deviation patterns rather than mean group differences, we consider that adding case–control analyses would not increase the interpretability of the central findings. However, if the reviewer or the editors still consider this would be a valuable addition to this paper, we will provide it.*

7. In the Classification Pipelines subsection, it is stated that ComBat-GAM and all preprocessing steps were applied in a cross-validated manner. It might be clarified whether this cross-validation concurs with the ten-fold cross-validation (with grid search) later discussed in the Hyperparameters optimization and performance estimation section, and extends also to the training and test folds.

*Thank you for the opportunity to clarify this point. Yes, the cross-validation applied to ComBat-GAM and all preprocessing steps fully coincides with the ten-fold cross-validation scheme (with grid search) described in the Hyperparameters Optimization and Performance Estimation section and extends to both training and test folds. Specifically, within each fold iteration, ComBat-GAM parameters were estimated exclusively on the training folds and then applied to both the training and corresponding held-out test fold. Hyperparameter optimization was likewise restricted to the training data within each cross-validation iteration. This procedure ensured that no information from the test folds leaked into model training or preprocessing. We have clarified this explicitly in the Methods section (lines 259-266).*

8. In Supplementary 1.8, further details on grid values and results appear to be in Table S4, and not S3.

*We apologize for this oversight, which we have corrected.*

9. In general, a pertinent question would be whether discrimination of AN and HC via brain morphology metrics offers any advantages over that of simply using BMI/weight, which would be a relatively more-accessible modality. This might be briefly discussed, possibly together with a simple analysis on the classification performance of BMI towards AN/HC.

*We thank the reviewer for raising this important point. We agree that BMI is a highly informative and easily accessible marker for distinguishing AN from HC in many clinical contexts – even though a diagnosis of AN require additional criteria. In the present study, however, classification based on BMI alone would be largely trivial, as markedly low BMI is part of the diagnostic definition of AN and was therefore embedded in the inclusion criteria for AN and the exclusion criteria for HC; consequently, AN and HC BMI distributions show minimal overlap. For this reason, we did not perform a BMI-only classification analysis, as it would not provide meaningful or independent information given our study design.*

*While the aim of brain morphology–based AN vs. HC classification was not to propose a substitute for BMI in routine diagnosis, using brain measures might become relevant in situations where differential diagnosis is challenging, such as atypical presentations* [6]*,* *apparent absence of other AN criteria due to poor insight or treatment ambivalence* [7] *or comorbid medical conditions* [8,9]*. For this reason, we believe that future machine-learning studies would benefit from explicitly including underweight HC or individuals with low BMI due to non-psychiatric causes, as this would allow a more direct assessment of the incremental value of brain morphology beyond BMI. However, assembling such samples requires careful clinical characterization to exclude eating disorders and to account for somatic causes of low weight* [10]*.*

*We have added a brief discussion of this point to the Discussion (lines 504-513).*


**Reviewer #2**: I thank the author team for a fascinating read on such a comprehensive mega-analysis evaluating morphological differences in those with anorexia nervosa and their subtypes. I not only commend the wide range of methodological approaches implemented in this study, but also the extensive array of available data utilised to address research questions. All feedback and suggestions may be found within the attached documentation.

The following manuscript provides a mega-analysis of neuroimaging data from 11 international sites to investigate structural differences in those with anorexia nervosa (AN) relative to healthy control. This mega-analysis utilised group-wise differences, evaluation of structural in relation to referential z-scores, and machine learning approaches to investigate brain morphology in AN and their subtypes. Findings confirm those seen in extant literature identifying widespread reductions in grey matter in those with AN relative to HCs, and could be accurately classified using machine learning approaches. This study uses multiple statistical techniques to investigate neuroanatomical nuances in those with AN, and is significantly strengthened by the large sample sizes available and subsequent study power. The methodological approach is clearly and transparently outlined, particularly the fine-tuning of their optimisation parameters for machine-learning-based classification. There are very minor issues regarding referencing, but more importantly I have concerns regarding BMI-based cut-off thresholds to distinguish between study groups and what implications this may have on morphological findings.

A significant proportion of neuroimaging research in anorexia nervosa include studies with limited sample sizes, and the extent and array of data provided within this mega-analysis is a considerable strength of this study, and provides enormous benefit towards the understanding of nuanced variations in brain structure in AN. Providing the authors can address methodological concerns, I have no doubt this study will make a significant contribution to the current literature. Suggestions to bolster this work alongside additional considerations are further outlined below.

*We thank the reviewer for this positive and encouraging assessment of our work. We appreciate the recognition of the scale of the mega-analysis as a key strength and have addressed all methodological concerns and additional suggestions in the revised manuscript.*

Introduction
1.      Minor: the following sentence requires a hyphen for ‘group level’, as well as a space between ‘subtypes’ and ‘[7]’: “In contrast to our previous group level meta-analysis, analyzing individual-level data enables examination of variability related to clinical AN subtypes[7].”      Introduction   P.4

*We thank the reviewer for noting this and have amended it accordingly.*

2.      To assist prospective readers, I’m wondering if the authors may be able to further elaborate on the distinction between the AN-BP subtype and bulimia nervosa. Recent literature reviewing AN subtypes has debated the classification and operationalisation of AN-BP and AN-R subtypes (Albracht-Schulte KD, Flynn L, Gary A, Perry CM, Robert-McComb JJ. The Physiology of Anorexia Nervosa and Bulimia Nervosa. In The Active Female: Health Issues throughout the Lifespan 2023 Feb 26 (pp. 95-117). Cham: Springer International Publishing.), as well as significant diagnostic crossover and transition between subtypes (Serra R, Di Nicolantonio C, Di Febo R, De Crescenzo F, Vanderlinden J, Vrieze E, Bruffaerts R, Loriedo C, Pasquini M, Tarsitani L. The transition from restrictive anorexia nervosa to binging and purging: a systematic review and meta-analysis. Eating and Weight Disorders-Studies on Anorexia, Bulimia and Obesity. 2022 Apr;27(3):857-65.).
I think this debate is important for the author team to acknowledge, and has additional implications on study hypotheses associated with structural morphology of the brain.    Introduction    P.4

*We thank the reviewer for this important suggestion. We have expanded the Introduction (lines XX–XX) to further clarify the diagnostic distinction between AN-BP and bulimia nervosa, and to explicitly acknowledge the ongoing debate regarding the classification and operationalization of AN subtypes, including diagnostic crossover and transition. We agree that this debate has direct implications for hypotheses concerning structural brain differences; accordingly, we now state upfront in the Introduction (lines 119-123) that the present study may inform this discussion. In addition, we have expanded the interpretation of our findings in the Discussion (lines 533-541) to explicitly consider the absence of marked structural differences between subtypes in the context of this nosological debate.*

3.      Similar to the comment above, I think it is pertinent to touch upon previous literature aiming to investigate brain morphological or functional distinctions between AN subtypes (e.g., Yonezawa H, Otagaki Y, Miyake Y, Okamoto Y, Yamawaki S. No differences are seen in the regional cerebral blood flow in the restricting type of anorexia nervosa compared with the binge eating/purging type. Psychiatry and clinical neurosciences. 2008 Feb;62(1):26-33.), and how the availability and richness of data present in this study may be more suitable to comprehensively investigate these nuances.
I note that the authors have begun to touch upon this at the bottom of page four within their introduction, but I think that discussion of previous literature would further strengthen the study rationale.
        Introduction    P.4

*We thank the reviewer for this useful reference, which we have integrated into the Introduction (lines 127-132).*

4.       Minor: There is an issue with plurality in the following text: “(e.g., cortical thickness (CT) in specific brain region)”      Introduction    P.5

*We thank the reviewer for noting this and have amended it accordingly.*

Methods
1.      Minor: As the authors have already abbreviated ‘the ENIGMA Eating Disorder Working Group’ to read as ‘The ENIGMA ED working group’ within the Introduction, I would suggest they remain consistent in use of the abbreviation within the methods and subsequent text.    Methods – Study samples        P.6

*We thank the reviewer for noting this and have amended it accordingly.*

2.      Could the authors elaborate on the BMI cut-offs used for adults within their inclusion and exclusion criteria? ICD-11 classifications characterise AN as having a BMI falling below 18.5 kg/m2, while DSM-5 classifications include BMI ranges, which mark AN severity as mild (BMI ≥ 17 kg/m2), moderate (BMI 16–16.99 kg/m2), severe (BMI 15–15.99 kg/m2) or extreme (BMI < 15 kg/m2; Himmerich H, Treasure J. Anorexia nervosa: diagnostic, therapeutic, and risk biomarkers in clinical practice. Trends in Molecular Medicine. 2024 Apr 1;30(4):350-60.).
Considering the present range of <17.5 for AN falls between these classifications, it would benefit readers for the authors to rationale this cut-off. This includes elaborating upon including adults with a BMI below a healthy range within their HC group. I hope the authors have a solidified rationale for this decision, considering a significant portion of neuroanatomical research into AN has failed to accurately distinguish brain atrophy associated with malnutrition from AN-specific morphological changes.   Methods – Study samples         P.6

*We thank the reviewer for pointing out this discrepancy and apologize for the confusion it may have caused. In the present study, we consistently applied the ICD-11 criterion, using a BMI ≤ 18.5 kg/m² as the upper limit for inclusion in the AN group. This threshold reflects current diagnostic standards, captures the full clinically relevant spectrum of underweight associated with AN, and is commonly used in contemporary research to ensure comparability across studies* [11]*. The value of < 17.5 reported elsewhere was inadvertent and has been corrected in the Methods (lines 172-177) and Supplement. For HC, we included participants with BMI ≥ 17.5 kg/m². Although this range includes some underweight individuals, all HC were screened as healthy eaters without evidence of an eating disorder or other underlying conditions. In our sample, 2.7% (12/449) of adult HC (>18 years) were mildly underweight (BMI < 18.5 kg/m²), which is broadly consistent with population-level estimates for European and North American countries (https://www.who.int/data/gho/data/indicators/indicator-details/GHO/prevalence-of-underweight-among-adults-bmi-18-%28crude-estimate%29-%28-%29). Therefore, our rationale was to keep the HC sample representative of the general population rather than excluding individuals with BMI < 18.5 kg/m². Nevertheless, we agree that studies with BMI-matched control groups are important to disentangle brain alterations related to low body weight per se from AN-specific morphological changes. In line with this, a very recent study comparing brain structure in underweight HC, normal-weight HC, and AN for the first time, reported both BMI-related alterations (present in AN and underweight HC) and AN-specific alterations (present in AN but not underweight HC)* [1]*. We have added a sentence in the Methods to clarify our rationale (lines 177-183) and cited the above-mentioned study in the Discussion (lines 550-554).*

3.      I think the reader would benefit from a short, sentence-long description as to how AN subtypes were classified prior to referencing the Supplementary Methods.
        Methods – Sample of cases with subtype information (AN-R vs. AN-BP)      P.6

*The reviewer is right that this information would improve readability. We have explained in the revised manuscript that AN subtype was assessed at each site using standardized diagnostic interviews or expert clinical evaluations based on DSM-IV/DSM-5 or ICD-10 criteria and referred to the SM1.3 for more detailed information (lines 199-201).*

4.      Which MRI manufacturers and models were utilised for T1-weighted image acquisition across sites? Retrospectively, I can see that these are provided within Table S3, but can the authors make mention of this within the methods?
         Methods – Image acquisition and processing     P.7

*We thank the reviewer for this suggestion. We have now explicitly added information on MRI scanner vendors and models used for T1-weighted image acquisition to the Methods section (lines 207-210), with full details retained in Table S3.*

5.      Minor: The reference for Pomponio et al. (2020) is not numbered, and references Bahnsen et al. 2022 and Bernardoni et al. 2016 seem to use a different referencing style than the rest of the manuscript.        Methods – Group comparisons    P.7

*Thank you for spotting these inconsistencies, which we have amended.*

6.      Minor: Should CentileBrain be appropriately referenced at first mention?        Methods – Normative modeling    P.8

*We have added a reference for the CentileBrain initiative also at first mention.*

7.      Could the authors include mention of using 2-proportion z-tests to assess proportions of infranormal or supranormal z-scores between AN-BP and AN-R?    Methods – Normative modeling    P.8

*We apologize that this important detail was missing, and have added in the revised manuscript (lines 248-250).*

8.      Minor: Arold et al. (2023) should be appropriately numbered to remain consistent with referencing style.        Methods – Machine learning classification       P.8

*We thank the reviewer for noting this and have amended it accordingly.*

9.      Minor: Under ‘Explainable AI’, “AI” should be spelled out in full at first mention      Methods – Machine learning classification       P.10

*We thank the reviewer for spotting this and have amended it accordingly.*

Results
1.      Minor: the AN subtypes should be abbreviated in the following sentence for consistency: “The age difference in subtypes may reflect the more typical transition from the restricting to the binge-eating/purging subtype over time [14, 43, 44].”        Results - Demographics P.10

*We thank the reviewer for noting this and have amended it accordingly.*

2.      Minor: Please remain consistent with spelling of “Mean (SD)”; at times it is presented with a space before brackets (e.g., ‘Mean (SD)’), and during other times no space is present (e.g., ‘Mean(SD)’).         Results – Univariate comparisons        P.10

*We thank the reviewer for noting this and have applied the Mean (SD) notation consistently throughout the entire manuscript.*

3.       To provide readers with a bit of context, could the authors elaborate on what would generally be considered appropriate or high performance for classifier models, particularly in reference to ROC-AUC values? I think the provision of referential values would ease reader interpretation of these findings, particularly those less well-versed in machine learning techniques.    Results – Machine learning classification       P.14

*We thank the reviewer for this helpful suggestion. To facilitate interpretation, we have added brief contextual information in the Results section describing commonly used reference ranges for ROC–AUC values (e.g., chance-level at 0.5, values around 0.7–0.8 indicating acceptable discrimination, and values ≥0.8 reflecting strong discrimination). In addition, we now clarify the interpretation of PR–AUC values by explicitly relating them to the baseline defined by class prevalence, which varies across classification tasks (lines 377-381, lines 390-391).*

Discussion
1.      Minor: Is it necessary to re-introduce the abbreviations for cortical thickness, subcortical volume and cortical surface area within this section?
        Discussion      P.16

*We agree that re-introducing these abbreviations in this section is not necessary, and they have therefore been removed in the revised manuscript.*

2.      Minor: Walton et al. (2022) should be appropriately numbered, and the year should be included within the following portion of text: “the effect sizes were comparable (d=-0.55(0.21) vs. d=-0.57(0.20) in Walton et al.), while […]”    Discussion      P.16 & P.17

*We thank the reviewer for noting this and have amended it accordingly.*

3.      Is there extant literature that the authors are able to draw on regarding their interpretation of findings stemming from their analysis of z-scores from the CentileBrain group? Have other studies utilised this approach, and can the authors explicitly acknowledge this? The authors are making multiple interpretations in this section that, without reference to adequate evidence, borders on speculation rather than interpretation.   Discussion      P.17

*We agree that interpretation of CentileBrain-derived z-scores should be explicitly grounded in prior work. Normative modelling approaches that quantify subject-level deviations from an age- and sex-anchored reference distribution (commonly expressed as z-scores) have been used to characterize inter-individual heterogeneity in psychiatric neuroimaging and to show that case–control contrasts can obscure marked variability at the individual level* [12]*. There are multiple examples in the literature using normative models but here we focus on the CentileBrain model. We chose to apply models developed by the CentileBrain Initiative using the CentileBrain Platform (www.centilebrain.org) because the algorithms and covariates have been extensively benchmarked* [5]*, and are sex-specific which is a unique advantage in eating disorders where the sex distribution of the clinical population is substantially skewed towards females. The CentileBrain models have been applied to neuroimaging data of diverse psychiatric and neurological populations, with several studies already published or nearing publication* [13–15] *. In Ge et al., the utility of the CentileBrain models in first-episode psychosis was shown* [5]*, while in another study individuals at clinical high risk for psychosis were examined* [16]*. Other studies have identified heterogeneous deviations from the norm in psychiatric populations* [17,18]*, concluding that the “average patient” represents a largely non-informative construct* [18]*. When applying the CentileBrain normative model—which accounts for global measures such as mean cortical thickness—to anorexia nervosa and other conditions where global metrics differ substantially from healthy controls, care has to be taken when interpreting the results. For example, a supranormal z-score for the CT of a ROI does not indicate necessarily that a participant had increased CT in that ROI compared to an HC of the same age and sex, but that it had increased CT in that ROI compared to an HC of the same age and sex, but also same mean CT. Therefore, our findings of increased frequency of extreme z-scores compared to the norm, and of increased variability of z-scores in AN compared to HC need to be interpreted as follows. In underweight AN, where gray matter reductions are widespread relative to healthy controls (e.g., Walton et al.), we observed marked inter-individual variability, with some patients showing pronounced deviations in specific metrics while others were minimally affected. We have therefore expanded the Discussion to acknowledge prior normative-modelling studies in psychiatric populations that reached similar conclusions regarding inter-individual heterogeneity and the limited interpretability of group averages (lines 479-481).*

4.      The following text should fall within the methods or Supplementary Methods section, rather than within the discussion: “To address site-related variability, we implemented two complementary strategies. In the first, we applied ComBat-GAM to explicitly remove site effects prior to model training [33]. In the other, site correction was not performed; instead, we used a leave-site-out cross-validation (LSsO) strategy, ensuring that all test data came from sites unseen during training.” Discussion      P.17

*We thank the reviewer for this comment. The referenced text was repeated in the Discussion for readability; however, as suggested, it has now been removed from the Discussion to improve conciseness, with the methodological details retained in the Methods.*

Figures/Tables
1.      The following text should be included within the methods or Supplementary Methods, rather than within the figure legend: “To assess the importance of each feature for the classification, we computed the Pearson correlation coefficient between each feature and the machine learning-based risk score (Haufe et al., 2014)”. As a minor point, the reference should also be appropriately numbered as per the recommended style.    Figure 3.       P.15

*Thank you for this suggestion, which we have implemented as requested (lines …).*

Supplementary Methods
1.       The authors note here that participants were classified as having AN with a BMI <18.5, but this value is noted as 17.5 within the main manuscript. Could the authors clarify?  1.2 Study Criteria      P.29

*We thank the reviewer for pointing out this discrepancy and apologize for the confusion it may have caused. In the present study, we consistently applied the ICD-11 criterion, using a BMI ≤ 18.5 kg/m² as the upper limit for inclusion in the AN group. This threshold reflects current diagnostic standards, captures the full clinically relevant spectrum of underweight associated with AN, and is commonly used in contemporary research to ensure comparability across studies* [11]*. The value of < 17.5 reported elsewhere was inadvertent and has been corrected in the Methods (lines 172-177) and Supplement.*

2.      Table S3-Table S7 are missing appropriate abbreviations sections.       Table S3-Table S7       P.45-P.48

*We apologize for this oversight. Appropriate abbreviation sections have now been added to the legends of Tables S3–S7.*

3.      Similar to the supplementary tables, Figure S1-Figure S13 inconsistently display footnotes to clarify included abbreviations.   Figure S1-Figure S13    P.49-P.61

*Thank you for pointing this out. We agree that the use of footnotes explaining abbreviations across the supplementary figures was inconsistent. We have now revised Figures S1–S13 to ensure consistent inclusion of footnotes clarifying all abbreviations used.*

**Reviewer #4**:

Dear Authors and Editor,
Thanks for letting me read this interesting and well-written paper!
I need to directly state that I am not well experienced with imaging and the analyses conducted here, thus my review will be based on the more general parts of the paper and its design.

*We thank the reviewer for their careful reading of the manuscript and for the thoughtful and constructive comments. We appreciate the positive assessment of the clarity and overall quality of the paper.*

Major comments:

If I understand it correctly BMI was only taken into account in the subtype analyses, for which no differences were detected, and thus the findings could all be related to starvation/low BMI. Have the authors tested what happens when controling for BMI in the main analyses, i.e. AN vs HC? In line with this I also lack inclusion/sub-analyses of individuals recovered from AN, and if the number of studies including recovered patients are not enough (likely) for a meta-/megaanalyses this needs to be discussed and mentioned as a limitation.

*We thank the reviewer for this important point. The reviewer’s understanding is correct: BMI was regressed out in the subtype analyses, for which no significant differences were detected. In these analyses, our explicit aim was to identify potential neurobiological differences related to AN subtype per se, rather than differences driven by illness severity or degree of underweight, for which BMI serves as a proxy. Therefore, BMI was included as a covariate in the subtype analyses to reduce confounding by severity and to focus on subtype-specific effects. In contrast, we did not control for BMI in the main AN vs. HC analyses. Our rationale is that BMI is a defining clinical feature of anorexia nervosa and is therefore strongly confounded with diagnostic group membership, with acutely ill patients exhibiting substantially lower BMI than healthy controls (as per study design). Including BMI as a covariate in this context would require the model to extrapolate across largely non-overlapping BMI ranges, making it difficult to disentangle variance attributable to diagnosis from variance attributable to BMI, and rendering such analyses of questionable interpretability. Moreover, our goal in the AN vs. HC comparison was to replicate the analytical approach of prior large-scale ENIGMA studies, which likewise did not regress out BMI in case–control analyses. We have expanded the description of our rationale in the methods accordingly (lines 217-220, 225-231, 269-270).*

*At the same time, we agree that, as in previous studies, the present design does not allow a definitive disambiguation of effects related to the diagnosis of AN from effects related to starvation or low BMI per se. However, a recent study comparing both normal weight and underweight HC to AN found distinct AN-specific alterations in addition to alteration related to low BMI* [1]*. We have therefore explicitly added this point to the limitations section (lines 550-556) citing the aforementioned study.*

*With respect to recovered individuals, we agree that analyses including individuals recovered from AN would be highly informative. However, at present, only a small number of ENIGMA sites have collected sufficiently harmonized data on recovered patients, and data-sharing for this subgroup is still limited. Consequently, such analyses are not yet feasible within the ENIGMA framework. We have now added this as an additional limitation and note it as an important direction for future collaborative work (lines 553-556).*

I lack a discussion and speculation on how the specific regions in which differences are detected could relate to AN traits and phenotypes.

*‌Thank you for this suggestion. We agree that a more explicit discussion of how the regional pattern of effects may relate to AN traits/phenotypes would improve interpretability, and we have added such a paragraph to the Discussion (lines 447-466).*

*While acknowledging that anatomical specificity should be interpreted cautiously given that findings are widespread and previous study highlighted that reductions are more pronounced in more energy demanding regions* [19] *underlying the link with starvation mechanisms, we now briefly speculate on the most affected regions in our data. The largest CT effects were observed in the left superior and inferior parietal cortex (both d ≈ −0.96). Parietal regions are implicated in visuospatial processing and mental imagery and have repeatedly been discussed in relation to body representation/body image distortion and altered integration of multisensory information in AN* [20,21]*. For SA, the strongest effects were located in the bilateral transverse temporal gyri, a region typically associated with early auditory processing* [22]*; we note that links to core AN traits are necessarily speculative here and may reflect more general, distributed state effects rather than a specific auditory phenotype. For SV, the most pronounced reductions involved the bilateral thalamus, which is a central hub for sensory relay and cortico–subcortical integration* [23] *and has been reported as reduced in AN, with some evidence that thalamic measures relate to metabolic/endocrine indices (e.g., leptin) and BMI-related pathways* [24]*. Finally, we also comment on the few regions showing opposite (higher) CT in AN, specifically the left temporal pole and right entorhinal cortex (small effect sizes; d ≈ 0.15 and 0.13). Given the modest magnitude and the multiple-comparisons context, we treat these findings cautiously; nonetheless, these medial/anterior temporal regions have been implicated in socio-emotional* [25] *and memory-related processes* [26] *and may reflect compensatory mechanisms, or trait-like differences in subsets of individuals rather than starvation effects per se.*

Minor comments:
Different BMI criteria are stated in main text vs supplementary text, <17.5 vs <18.5. Or does the later only apply to patients in recovery phase? Please clarify.

*We thank the reviewer for pointing out this discrepancy and apologize for the confusion it may have caused. In the present study, we consistently applied the ICD-11 criterion, using a BMI ≤ 18.5 kg/m² as the upper limit for inclusion in the AN group. This threshold reflects current diagnostic standards, captures the full clinically relevant spectrum of underweight associated with AN, and is commonly used in contemporary research to ensure comparability across studies* [11]*. The value of < 17.5 reported elsewhere was inadvertent and has been corrected in the Methods (lines 172-177) and Supplement.*

I question the statement on p. 4t: "... and more severe medical complications due to the cycle of binge-eating and
purging."

*We thank the reviewer for this comment. We agree that the phrasing may be overly general and could be interpreted as implying a uniformly greater severity of medical complications in the binge-eating/purging subtype. To avoid potential overstatement, we have removed this sentence from the manuscript.*

**References**

1.	Wang Y, Wang Q, Chan RCK, Wang Y, Lei X, Qiu Q, et al. Distinct Structural Alterations in Cortical and Subcortical Regions in Females With Acute Anorexia Nervosa: A Cross‐Sectional MRI Study With BMI ‐Matched Healthy Controls. Int J Eat Disord. 2025; eat.24592. doi:10.1002/eat.24592

2.	Moreau CA, Ayrolles A, Ching CRK, Bonicel R, Mathieu A, Stordeur C, et al. Neuroimaging insights into brain mechanisms of early-onset restrictive eating disorders. Nat Ment Health. 2025;3: 780–788. doi:10.1038/s44220-025-00447-x

3.	Steegers C, Blok E, Lamballais S, Jaddoe V, Bernardoni F, Vernooij M, et al. The association between body mass index and brain morphology in children: a population-based study. Brain Struct Funct. 2021;226: 787–800. doi:10.1007/s00429-020-02209-0

4.	Van Eeden AE, Van Hoeken D, Hoek HW. Incidence, prevalence and mortality of anorexia nervosa and bulimia nervosa. Curr Opin Psychiatry. 2021;34: 515–524. doi:10.1097/YCO.0000000000000739

5.	Ge R, Yu Y, Qi YX, Fan Y, Chen S, Gao C, et al. Normative modelling of brain morphometry across the lifespan with CentileBrain: algorithm benchmarking and model optimisation. Lancet Digit Health. 2024;6: e211–e221. doi:10.1016/S2589-7500(23)00250-9

6.	Vo M, Golden N. Medical complications and management of atypical anorexia nervosa. J Eat Disord. 2022;10: 196. doi:10.1186/s40337-022-00720-9

7.	Gregertsen EC, Mandy W, Serpell L. The Egosyntonic Nature of Anorexia: An Impediment to Recovery in Anorexia Nervosa Treatment. Front Psychol. 2017;8: 2273. doi:10.3389/fpsyg.2017.02273

8.	Perera LAM, Chopra A, Shaw AL. Approach to Patients with Unintentional Weight Loss. Med Clin North Am. 2021;105: 175–186. doi:10.1016/j.mcna.2020.08.019

9.	Treasure J, Duarte TA, Schmidt U. Eating disorders. The Lancet. 2020;395: 899–911. doi:10.1016/S0140-6736(20)30059-3

10.	Bailly M, Thivel D, Isacco L, Verney J. Unique Energy Profile Associated with Persistent Thin Phenotype. Annu Rev Nutr. 2025;45: 65–91. doi:10.1146/annurev-nutr-111824-014837

11.	Himmerich H, Treasure J. Anorexia nervosa: diagnostic, therapeutic, and risk biomarkers in clinical practice. Trends Mol Med. 2024;30: 350–360. doi:10.1016/j.molmed.2024.01.002

12.	Marquand AF, Rezek I, Buitelaar J, Beckmann CF. Understanding Heterogeneity in Clinical Cohorts Using Normative Models: Beyond Case-Control Studies. Biol Psychiatry. 2016;80: 552–561. doi:10.1016/j.biopsych.2015.12.023

13.	Wang HR, ENIGMA MDD Working Group and ENIGMA PTSD Working Group, Ho TC. Childhood Maltreatment and Deviations from Normative Brain Structure: Results from 3,711 Individuals from the ENIGMA MDD and ENIGMA PTSD. Neuroscience; 2025. doi:10.1101/2025.06.04.657717

14.	Ditchfield C, Gates P, Domínguez D JF, Dhillon HM, Dore V, Wilson C, et al. Cognitive impairment and brain atrophy in patients with newly diagnosed aggressive lymphoma undergoing standard chemotherapy: a normative analysis. Front Neurol. 2025;16: 1601459. doi:10.3389/fneur.2025.1601459

15.	Martins D, Cai Z, Mariani N, Borsinni A, Mondelli V, Eiff B, et al. Molecular, cellular and network mapping of brain structural deviations in patients with Post-COVID19 syndrome. Neuroscience; 2026. doi:10.64898/2026.01.12.699045

16.	ENIGMA Clinical High Risk for Psychosis Working Group, Allen P, Baldwin H, Bartholomeusz CF, Chee MW, Chen X, et al. Normative Modeling of Brain Morphometry in Clinical High Risk for Psychosis. JAMA Psychiatry. 2024;81: 77. doi:10.1001/jamapsychiatry.2023.3850

17.	Haukvik UK, Wolfers T, Tesli N, Bell C, Hjell G, Fischer-Vieler T, et al. Individual-level deviations from normative brain morphology in violence, psychosis, and psychopathy. Transl Psychiatry. 2025;15: 118. doi:10.1038/s41398-025-03343-1

18.	Wolfers T, Doan NT, Kaufmann T, Alnæs D, Moberget T, Agartz I, et al. Mapping the Heterogeneous Phenotype of Schizophrenia and Bipolar Disorder Using Normative Models. JAMA Psychiatry. 2018;75: 1146. doi:10.1001/jamapsychiatry.2018.2467

19.	Bahnsen K, Bernardoni F, King JA, Geisler D, Weidner K, Roessner V, et al. Dynamic Structural Brain Changes in Anorexia Nervosa: A Replication Study, Mega-analysis, and Virtual Histology Approach. J Am Acad Child Adolesc Psychiatry. 2022; S0890856722001836. doi:10.1016/j.jaac.2022.03.026

20.	Brizzi G, Sansoni M, Di Lernia D, Frisone F, Tuena C, Riva G. The multisensory mind: a systematic review of multisensory integration processing in Anorexia and Bulimia Nervosa. J Eat Disord. 2023;11: 204. doi:10.1186/s40337-023-00930-9

21.	Gaudio S, Wiemerslage L, Brooks SJ, Schiöth HB. A systematic review of resting-state functional-MRI studies in anorexia nervosa: Evidence for functional connectivity impairment in cognitive control and visuospatial and body-signal integration. Neurosci Biobehav Rev. 2016;71: 578–589. doi:10.1016/j.neubiorev.2016.09.032

22.	Khalighinejad B, Patel P, Herrero JL, Bickel S, Mehta AD, Mesgarani N. Functional characterization of human Heschl’s gyrus in response to natural speech. NeuroImage. 2021;235: 118003. doi:10.1016/j.neuroimage.2021.118003

23.	Guillery RW, Sherman SM. Thalamic Relay Functions and Their Role in Corticocortical Communication. Neuron. 2002;33: 163–175. doi:10.1016/S0896-6273(01)00582-7

24.	Wronski M-L, Gronow F, Schlömer J, Bernardoni F, Geisler D, Doose A, et al. Structural alterations of thalamic nuclei and their associations with leptin levels in patients with anorexia nervosa. Prog Neuropsychopharmacol Biol Psychiatry. 2025;136: 111248. doi:10.1016/j.pnpbp.2025.111248

25.	Herlin B, Navarro V, Dupont S. The temporal pole: From anatomy to function—A literature appraisal. J Chem Neuroanat. 2021;113: 101925. doi:10.1016/j.jchemneu.2021.101925

26.	Ekstrom AD, Hill PF. Spatial navigation and memory: A review of the similarities and differences relevant to brain models and age. Neuron. 2023;111: 1037–1049. doi:10.1016/j.neuron.2023.03.001
