# Supplementary material for: Brain morphology in Anorexia Nervosa and its subtypes: A multi-cohort study of individual participant data
Source: PLoS Med. 2026 May 20;23(5):e1004809. doi: 10.1371/journal.pmed.1004809 (PMC13215615; doi:10.1371/journal.pmed.1004809)
Supplement: Attachment — Submitted filename: Bernardoni_et_al_Response2EditorsReviewers.docx [file pmed.1004809.s007.docx]

**Requests from Editors**

*Dear Dr. Bernardoni,*

*Thank you very much for re-submitting your manuscript "Brain morphology in Anorexia Nervosa and its subtypes: Mega-Analysis, Normative Modelling, and Machine Learning Approaches" (PMEDICINE-D-25-03733R2) for review by PLOS Medicine.*

*I have discussed the paper with my colleagues and the academic editor and it was also seen again by 4 reviewers. I am pleased to say that provided the remaining editorial and production issues are dealt with we are planning to accept the paper for publication in the journal.*

*The remaining issues that need to be addressed are listed at the end of this email. Any accompanying reviewer attachments can be seen via the link below. Please take these into account before resubmitting your manuscript.*

**Response:** Thank you very much for the encouraging assessment of our revised manuscript. We are grateful to you, the Academic Editor, and the reviewers for the time and care invested in evaluating our work. We appreciate the opportunity to address the remaining editorial and production issues, and we have revised the manuscript accordingly. Below, we provide a point-by-point response to each of the issues raised and indicate the corresponding changes made in the manuscript and supporting information.

*GENERAL EDITORIAL REQUESTS
Please note that some of the following requests may not apply to your manuscript.
"* At this stage, we ask that you include a short, non-technical Author Summary of your research to make findings accessible to a wide audience that includes both scientists and non-scientists. The Author Summary should immediately follow the Abstract in your revised manuscript. This text is subject to editorial change and should be distinct from the scientific abstract. Ideally each sub-heading should contain 2-3 single sentence, concise bullet points containing the most salient points from your study. In the final bullet point of ‘What Do These Findings Mean?’ Please include the main limitations of the study in non-technical language. Please see our author guidelines for more information:* [*https://journals.plos.org/plosmedicine/s/revising-your-manuscript#loc-author-summary*](https://journals.plos.org/plosmedicine/s/revising-your-manuscript#loc-author-summary)*."*

**Response:** We have provided an Author Summary according to the PLOS Medicine guidelines in the revised manuscript.

** Please confirm that your title complies with PLOS Medicine's style. Your title must be nondeclarative and not a question. It should begin with main concept if possible. "Effect of" should be used only if causality can be inferred, i.e., for an RCT. Please place the study design ("A randomized controlled trial," "A retrospective study," "A modelling study," etc.) in the subtitle (ie, after a colon).
I would strongly advise you to modify the title of your study to adhere to PLOS title guidelines. As an example you can instead use: "Brain morphology in Anorexia Nervosa and its subtypes: A multi-cohort study of individual participant data". This suggestion is not intended to be prescriptive, and you are encouraged to revise it as you see fit.*

**Response:** Thank you for this helpful suggestion. We have adopted your proposed title and revised the manuscript accordingly to align with the PLOS Medicine title guidelines.

** Please confirm that your abstract complies with our requirements, including format (three sections: Background, Methods and Findings, and Conclusions) and providing all the information relevant to this study type* [*https://journals.plos.org/plosmedicine/s/submission-guidelines#loc-abstract*](https://journals.plos.org/plosmedicine/s/submission-guidelines#loc-abstract) ** Please ensure that the Introduction ends with a clear description of the study question or hypothesis.
* Please ensure that all abbreviations are defined at first use throughout the text.
* Please confirm that all numbers presented in the abstract are present and identical to numbers presented in the main manuscript text.
* Please ensure that the study is reported according to the STROBE guideline, and include the completed STROBE checklist as Supporting Information.  Please add the following statement, or similar, to the Methods: "This study is reported as per the Strengthening the Reporting of Observational Studies in Epidemiology (STROBE) guideline (S1 Checklist)."

The STROBE guideline can be found here:* [*http://www.equator-network.org/reporting-guidelines/strobe/*](http://www.equator-network.org/reporting-guidelines/strobe/) *When completing the checklist, please use section and paragraph numbers, rather than page numbers.

While STROBE is recommended for observational studies, authors may additionally follow or substitute other relevant reporting guidelines where more appropriate to the specific study design or analytical approach, provided this is clearly stated and justified.*

**Response:** We have carefully reviewed the manuscript throughout to ensure compliance with these requirements. In particular, we checked the text for wording and formatting issues highlighted in the editorial comments and revised the manuscript accordingly where needed. In particular, we have edited the header of the relevant abstract section from “Introduction” to “Background” to conform to the journal’s format. We have also included the completed STROBE checklist as a separate document, indicating the paragraphs in which the relevant reporting items are addressed in the manuscript.

*GENERAL
* Please review your text for claims of novelty or primacy (e.g. 'for the first time') and remove this language. In addition, please check that any use of statistical terms (such as trend or significant) are supported by the data, and if not please remove them.*

**Response:** We have removed claims of novelty (e.g. in the abstract and in the discussion). In addition, we checked the use of statistical terms throughout the manuscript and ensured that terms such as “significant” or “trend” were used only where supported by the data and corresponding statistical results.

** In the last sentence of the Abstract Methods and Findings section, please describe the main limitation(s) of the study's methodology.*

**Response:** We have added such a sentence as suggested to explain that the main limitations are related to the study design which does not allow definitive disambiguation between neurobiological differences related to AN and effects of starvation or low BMI in the AN vs. HC analyses. The absence of findings in the subtype comparisons also does not rule out brain structural differences between AN subtypes that might be detectable with other modalities or machine-learning algorithms.

** In the abstract, please include the important dependent variables that are adjusted for in the analyses.*

**Response:** We have checked that this was the case.

** Please remove the 'conclusions' subheading from the discussion. Please also remove any other subheadings from the discussion.*

**Response:** We have removed all subheadings from the discussion.

*"* Statistical reporting: Please revise throughout the manuscript, including tables and figures.
- Please report statistical information as follows to improve clarity for the reader ""22% (95% CI [13,28]; p</=)"".*
*- Please separate upper and lower bounds with commas instead of hyphens as the latter can be confused with reporting of negative values.
- Please repeat statistical definitions (HR, CI etc.) for each set of parentheses."*

**Response:** We have revised the manuscript throughout, including the tables and figures, to comply with these reporting requirements. In particular, we now report 95% CIs for effect sizes in the univariate comparisons, use commas rather than hyphens to separate confidence interval bounds, and repeat statistical definitions within parentheses where appropriate. For the machine-learning analyses based on nested cross-validation, we did not add conventional 95% CIs, because such intervals are not straightforwardly defined from repeated cross-validation estimates due to dependence between folds. Instead, we report performance across outer test folds/repeats together with permutation-based significance testing.

** In the abstract, please include the important dependent variables that are adjusted for in the analyses.*

**Response:** We edited the abstract to specify the key covariates included in the analyses. In the AN vs. HC analyses, models were adjusted for age, whereas in the comparisons between AN restricting and binge-eating/purging subtypes, models were additionally adjusted for BMI..

*FUNDING STATEMENT
* The funding statement should include: specific grant numbers, initials of authors who received each award, URLs to sponsors’ websites. Also, please state whether any sponsors or funders (other than the named authors) played any role in study design, data collection and analysis, the decision to publish, or preparation of the manuscript. If they had no role in the research, include this sentence: “The funders had no role in study design, data collection and analysis, decision to publish, or preparation of the manuscript.”
*Please add the relevant URL for each funder.*

**Response:** We have checked that the funding statement complies with these requirements and added the URLs to sponsors’ websites.

*COMPETING INTERESTS STATEMENT
* All authors must declare their relevant competing interests per the PLOS policy, which can be seen here:* [*https://journals.plos.org/plosmedicine/s/competing-interests*](https://journals.plos.org/plosmedicine/s/competing-interests) *For authors with ties to industry, please indicate whether any of the interests has a financial stake in the results of the current study.

DATA AVAILABILITY
"* PLOS Medicine requires that the de-identified data underlying the specific results in a published article be made available, without restrictions on access, in a public repository or as Supporting Information at the time of article publication, provided it is legal and ethical to do so. Please see the policy at http://journals.plos.org/plosmedicine/s/data-availability" \t "_blank and FAQs at*
[*http://journals.plos.org/plosmedicine/s/data-availability#loc-faqs-for-data-policy*](http://journals.plos.org/plosmedicine/s/data-availability#loc-faqs-for-data-policy)  *"
"* The Data Availability Statement (DAS) requires revision. For each data source used in your study:
a) If the data are freely or publicly available, note this and state the location of the data: within the paper, in Supporting Information files, or in a public repository (include the DOI or accession number).
b) If the data are owned by a third party but freely available upon request, please note this and state the owner of the data set and contact information for data requests (web or email address). Note that a study author cannot be the contact person for the data.
c) If the data are not freely available, please describe briefly the ethical, legal, or contractual restriction that prevents you from sharing it. Please also include an appropriate contact (web or email address) for inquiries (again, this cannot be a study author)."
*Please provide third party contact information for data access; authors or the principal investigator cannot be used as the contact person.
* Thank you for agreeing to make your data available. At this time, please provide the link to the data repository and accession numbers required for access. Please check that the link you provided is correct as I cannot access it. Please also archive the code and provide the relevant link, eg via Zenodo.*

**Response:** We have checked that the above-mentioned statements comply with the requirements of PLOS Medicine. In particular, we revised the Data Availability Statement to include, for each site, contact information for the relevant ethics committee or institutional body that can be contacted regarding data access and data-sharing inquiries. We also corrected the repository link and ensured that it is accessible to individuals who are not project owners: https://osf.io/bvryd/overview?view_only=8e0839869b83400f93418ed24f8237bf

*ETHICS AND CONSENT
* Please provide the name(s) of the institutional review board(s) that provided ethical approval.
* Please, include a paragraph in the main methods where you clearly state what type of consent was obtained and a short description of your ethics approval, which then cites the supplementary material for further information.*

**Response:** We have added in the Methods a sentence (lines 208-210) stating that all participating cohorts received approval from their local institutional review boards or ethics committees, and all participants provided written informed consent. There we also cite the supplementary material for further information including the approval numbers.
*FIGURES
* Please define all elements of box plots in the figure caption - center line, box limits and whiskers.
* Please provide titles and legends for all figures and tables (including those in Supporting Information files). Please define all acronyms used in each figure or table in its corresponding legend.
* Please convert any pie charts to another data representation for example a table, or other type of graph.
* Please show graph axes beginning at zero. If this is not possible, please show a break in the axis.
* Please consider avoiding the use of red and green in order to make your figure more accessible*

**Response:** We have revised the figures, tables, and corresponding legends throughout the manuscript and Supporting Information to address these points. In particular, we defined the elements of the box plots in the relevant figure captions, provided titles and legends for all figures and tables, and defined acronyms in the corresponding legends. We also revised the graphs to ensure that axes begin at zero.

Regarding pie charts, we have retained the pie charts in Supplementary Figures S1 and S3, as the same information is also provided in tabular form in Supplementary Tables S1 and S2.

We thank the editors for this suggestion. We understand that the main accessibility concern is the use of red and green within the same figure to encode different information. In our revised submission, such use of red and green is limited to figures in the Supplementary Materials. If the editors consider further revision necessary, we would of course be happy to make these changes, although regenerating these figures will require additional time.

*Acknowledgments
*Please separate the Acknowledgements and Disclosures section into two individual sections.*

**Response:** These sections have been separated as requested.

*Comments from Reviewers:
Reviewer #1: We thank the authors for addressing our previous concerns.
Reviewer #2: Manuscript Title: Brain morphology in Anorexia Nervosa and its subtypes: Mega-Analysis, Normative Modelling, and Machine Learning Approaches
The following revised manuscript provides a mega-analysis of neuroimaging data from 11 international sites  to investigate structural differences in those with anorexia nervosa (AN) relative to healthy controls. The authors have comprehensively attended to the points brought up by all reviewers. My previous review of this manuscript comprised of potential concerns regarding the classification of AN subtypes, descriptions of group-based BMI cut-offs and interpretation of CentileBrain-derived z scores, which I believe have been adequately addressed.
I only have minor grammar- or punctuation-based suggestions for this work, as outlined below:
1.      Methods (Study samples; P.10; Lines 279-280): Minor - I think there may be a plurality issue in the following sentence and would recommend the following:
"We jointly optimized the model hyperparameters (e.g., number of PCA components) via a stratified (i.e., keeping the group proportions constant across partitions) ten-fold cross-validated grid search (Table S4)"
2.      Methods (Hyperparameters optimization and performance estimation; P.11; Lines 295-296): Minor - The following sentence requires a space between the text and bracket:
"To better approximate such scenarios, we also used Leave-Site(s)-Out cross-validation(LSsO, Nunes et al.2018)"
Reviewer #3: The current revised version provided new information in a high-quality way. I have no more questions.
Reviewer #4: All the comments raised by me and the other reviewers have been responded to and taken into account in a satisfying manner.*

**Response:** We thank the Reviewers for their positive evaluation of the revised manuscript and for noting that the previous concerns were addressed satisfactorily. We are grateful for their careful re-evaluation of the paper. We also thank Reviewer 2 for the additional minor language and formatting suggestions, which we have implemented in the revised manuscript.
