# Supplementary material for: Brain morphology in Anorexia Nervosa and its subtypes: A multi-cohort study of individual participant data
Source: PLoS Med. 2026 May 20;23(5):e1004809. doi: 10.1371/journal.pmed.1004809 (PMC13215615; doi:10.1371/journal.pmed.1004809)
Supplement: Attachment — Submitted filename: Bernardoni_et_al_ResponseEditors.docx [file pmed.1004809.s008.docx]

**Response to the Editors**

Thank you very much for your careful review of our revised manuscript. We appreciate the opportunity to address the remaining editorial issues. We have carefully revised the manuscript, figures, tables, Supporting Information, and online submission metadata as requested. Below, we provide a point-by-point response to each comment and indicate the corresponding changes made in the revised files.

** Data Availability Statement (DAS). The DAS statement is different between manuscript files and metadata. Please make sure that all metadata exactly match statements/text within the manuscript files. Regarding the DAS statement please use the one from the manuscript files*
** Thank you for updating the OSF link, which is now accessible. Please, archive the code and provide the relevant DOI link as well.*

We updated the analysis code (which was needed to generate the new supplementary figures, see below), made it publicly available under a new OSF link, registered it, and added the corresponding DOI to the Data Availability Statement. We also copied this statement into the online submission metadata.

** Figures S2 and S4:Please explain why some p-values = 0. If p-value numbers are too small, use p<0.001. I also suggest adding an asterisk when differences are significant for ease of readability (please define the asterisk in the legend if you proceed with the recommendation).*

Thank you for these suggestions. We changed p = 0.0001 to p < .001 in Figures S2 and S4, as requested. We also added asterisks where differences are significant to improve readability and defined the asterisks in the legend.

** Please revise the whole manuscript and define all abbreviations in text, figure legends and tables. Especially for figure legends and tables, content should be self-contained; therefore all abbreviations must be defined. Figures 1 and 2 for example are missing relevant abbreviations (AN, HC, CT, GLM).*

We apologize that some abbreviations were not defined. We revised the manuscript throughout to ensure that all abbreviations are defined where needed. In particular, we defined the abbreviations AN, HC, CT, and GLM in all relevant figure captions.

** Figure 1: the legend is not detailed enough. Please provide more details to facilitate readers, especially relating to significance and colouring presentation.*

Thank you for this suggestion. The caption to Figure 1 has been expanded to improve clarity.

** Figure 3 lacks a title.*

We apologize that this was missing. We have added the title: Feature importances for the anorexia nervosa (AN) vs. healthy controls (HC) classification.

** The introduction should end with a clear description of the study question and hypothesis. Currently, the introduction ends with a short abstract-like format. Please substitute this (Line 175-201) with a small paragraph clearly stating the question and hypothesis. If you wish to keep the format as is, please make sure to conclude the introduction with a clear description of the study question and hypothesis.*

Thank you for these suggestions. We have decided to keep the format as is, but we have added a paragraph with a clear description of the study questions and hypotheses (lines 203-215).

** Suggested changes in the paragraph "demographics" in the methods. It is slightly dense. I suggest using "across sites" instead of "overall", removing "instead" (line 358, since it does not contradict the previous statement) and "only" (line 359).*

Thank you for these corrections. We have implemented them as suggested.
